# A computational analysis of the long-term regulation of arterial pressure

**DOI:** 10.12688/f1000research.2-208.v2

**Published:** 2013-12-06

**Authors:** Daniel A. Beard, Klas H. Pettersen, Brian E. Carlson, Stig W. Omholt, Scott M. Bugenhagen

**Affiliations:** 1 Molecular and Integrative Physiology, University of Michigan, Ann Arbor, MI 48105, USA; 2 Department of Mathematical and Technological Sciences, Norwegian University of Life Science, Oslo, Norway; 3 Department of Physiology, Medical College of Wisconsin, Milwaukee, WI 53226, USA; 4 Cardiac Exercise Research Group, Department of Circulation and Medical Imaging, NTNU Norwegian University of Science and Technology, Trondheim, Norway

## Abstract

The asserted dominant role of the kidneys in the chronic regulation of blood pressure and in the etiology of hypertension has been debated since the 1970s. At the center of the theory is the observation that the acute relationships between arterial pressure and urine production—the acute pressure-diuresis and pressure-natriuresis curves—physiologically adapt to perturbations in pressure and/or changes in the rate of salt and volume intake. These adaptations, modulated by various interacting neurohumoral mechanisms, result in chronic relationships between water and salt excretion and pressure that are much steeper than the acute relationships. While the view that renal function is the dominant controller of arterial pressure has been supported by computer models of the cardiovascular system known as the “Guyton-Coleman model”, no unambiguous description of a computer model capturing chronic adaptation of acute renal function in blood pressure control has been presented. Here, such a model is developed with the goals of: 1. representing the relevant mechanisms in an identifiable mathematical model; 2. identifying model parameters using appropriate data; 3. validating model predictions in comparison to data; and 4. probing hypotheses regarding the long-term control of arterial pressure and the etiology of primary hypertension. The developed model reveals: long-term control of arterial blood pressure is primarily through the baroreflex arc and the renin-angiotensin system; and arterial stiffening provides a sufficient explanation for the etiology of primary hypertension associated with ageing. Furthermore, the model provides the first consistent explanation of the physiological response to chronic stimulation of the baroreflex.

## Introduction

Theoretical analysis and observations of the control of blood volume and salt content by the kidneys has led to the hypothesis that arterial pressure is determined in the long-term (over time scales of days or more) by the balance between the level of salt intake and the acute relationship between pressure and salt excretion by the kidneys. In fact, it is stated that the renal pressure/volume control system adjusts arterial pressure with “infinite gain” and that the renal function curve and rate of salt and water intake are the “two sole determinants of the long-term arterial pressure” ^1^. Yet while it is certain that at any steady level of arterial pressure the rates of salt and water intake and excretion are balanced, there is considerable debate over how this balance is achieved, and thus what are the long-term determinants of arterial pressure ^2– 5^.

One viewpoint is that the acute relationship between pressure and salt excretion—the acute pressure-natriuresis mechanism—represents a physiological input-output relationship and that alterations to this mechanism underlie most (if not all) chronic changes in pressure ^1, 6^. While competing ideas identify the nervous system and its influence on the heart and vasculature as the primary long-term controllers of arterial pressure ^3, 4^, the hypothesis that long-term control of blood pressure is achieved through the renal pressure-diuresis represents the dominant thinking in the field ^7^.

The most widely recognized model of long-term blood pressure control is the model developed over several decades by Guyton and colleagues. The 1972 realization of the model ^6^ invokes approximately 160 variables, and several hundred adjustable parameters ^8^. More recent versions of the model involve many thousands of variables and tens of thousands of parameters ^9, 10^. While several realizations of the Guyton model are or have been disseminated as computer programs, human readable expositions of the various versions of the model are lacking. Moreover, there is no published report of any of these models in which the governing equations are defined, the parameter values are provided, the data that were used for model identification are reported, and the model identification process and results are reported. Indeed, it is unlikely if not impossible that even the relatively simple 1972 version of the model was formally identified. These facts unfortunately make this seminal work largely impenetrable and possibly irreproducible. Most critically, the Guyton-Coleman model is based on the assertion that arterial blood pressure is, under all circumstances, controlled primarily by the kidney ^11^. Therefore the Guyton-Coleman model cannot be used to explore alternative hypotheses.

In the Systems Approach for PHysiological Integration of Renal, cardiac and respiratory functions (SAPHIR) project, Thomas *et al.* ^8^ aimed to develop and disseminate a core “model of human physiology targeting the short- and long-term regulation of blood pressure, body fluids and homeostasis of the major solutes” including “the main regulatory sensors (baro- and chemoreceptors) and nervous (autonomic control) and hormonal regulators (antidiuretic hormone, aldosterone and angiotensin)”. The project goal is to, for the first time, provide an open-source transparent model integrating these systems. In 2008, the SAPHIR group published a core model of circulation and volume exchange across fluid compartments that is derived from the Guyton models, consisting of 20 equations and perhaps less than 100 parameters ^8^. This model does not account for adaptation of the acute pressure-diuresis/natriuresis function to chronic changes in arterial pressure or volume/salt loading. Thus, it does not capture the concept of the chronic renal function curve, which is thought to be of fundamental importance to the long-term regulation of arterial pressure. Recently Averina *et al.* ^4^ reported a mathematical model of long-term control of arterial pressure that captures the concept of chronic adaptation of pressure-natriuresis and, to our knowledge, represents the only published model incorporating the concept of adaptation of the acute pressure-natriuresis relationship in response to changes in salt/volume loading. Thus, the model of Averina *et al.* represents the model of record of regulation of blood pressure accounting for the chronic renal function curve. Yet, this model was developed to illustrate that the chronic renal function curve need not represent a physiological input-output mechanism, and to introduce an alternative description of the long-term regulation of arterial pressure and the etiology of salt- and angiotensin-dependent hypertension.

Similar to the goals of the SAPHIR project, we developed a mathematical model of the Guyton concept of long-term control of arterial pressure, analysed its behavior and compared its predictions to experimental observations. Similarly, an open-source transparent model integrating these systems is provided. The approach pursued here differs from that of Thomas and colleagues in that while the SAPHIR project explicitly centers on translating the components of the Guyton model into an explicitly defined core model, our goal is to develop a model from scratch in which all model components and associated data are formally identified based on experimental data. Furthermore, given the demands of model identification and validation, our goals are necessarily more narrowly constrained than those of the SAPHIR project and of the original Guyton-Coleman models. In our study, a model is constructed in the spirit of the Guyton-Coleman models, adopting a practical approach to data-driven phenomenological representations of physiological systems, rather than aiming for physical and mechanistic realism.

Specifically, we have developed a model of the long-term control of arterial pressure that captures the Guyton concept of pressure-diuresis/natriuresis as physiological input-output relationships. The model is designed to meet the following criteria: 1. The model accounts for the effects of the baroreflex on the circulation, the heart, and the kidney, and the effects of the renin-angiotensin system on the circulation, the heart, and the kidney; 2. The model is explicitly documented for definition and reproducibility: all model equations are reported herein and justified based on experimental data; 3. Parameter values are reported and justified: All parameter values are estimated by comparing model simulations to measured data. The identified model is used to probe physiological mechanisms underlying the chronic renal function curve, to provide insight into how chronic stimulation of the baroreflex leads to chronic reductions in arterial pressure, and to generate hypotheses regarding the etiology of primary hypertension.

## Methods

### Model Components 1: Aorta/large-artery mechanics

Based on the simple approximation of a thin-walled cylinder, the strain *ε* in the aorta is computed as a function of volume *V _Ao_*

$$\varepsilon =\frac{d_{A}}{d_{0}}={\left(\frac{V_{Ao}}{V_{0}}\right)}^{1/2},\;\;\;\;\;(1)$$

where *V*_0_ is a parameter representing the unstressed volume, and *d _A_*/ *d*_0_ is the ratio of diameter to unstressed diameter. (Here *ε* is defined to be equal to 1 in the unstressed state when *d _A_* = *d*_0_). The aortic pressure-volume relationship is assumed to be governed by

$$P_{Ao}=\left(V_{Ao}-V_{sAo}\right)/C_{Ao}\;,\;\;\;\;\;(2)$$

where *C _Ao_* represents an acute compliance and *V _sAo_*( *t*) accounts for creep mechanics of aortic wall, simulated according to

$$\begin{array}{l} \tau_{cAo}\frac{dV_{sAo}}{dt}=V_{sAo}^{\infty }-V_{sAo}\\ V_{sAo}^{\infty }=\left(1-\frac{C_{Ao}}{C_{\infty }}\right)V_{Ao}=\gamma_{Ao}V_{Ao},\;\;\;\;\;(3) \end{array}$$

where *τ _cAo_* is the time constant of stress relaxation and *C _Ao_*/ *C*_∞_ is the ratio of acute to effective chronic compliance of the vessel. ( Equation (2) and Equation (3) represent an alternative equivalent formulation of the standard linear model of vessel mechanics).

**Figure 1.**
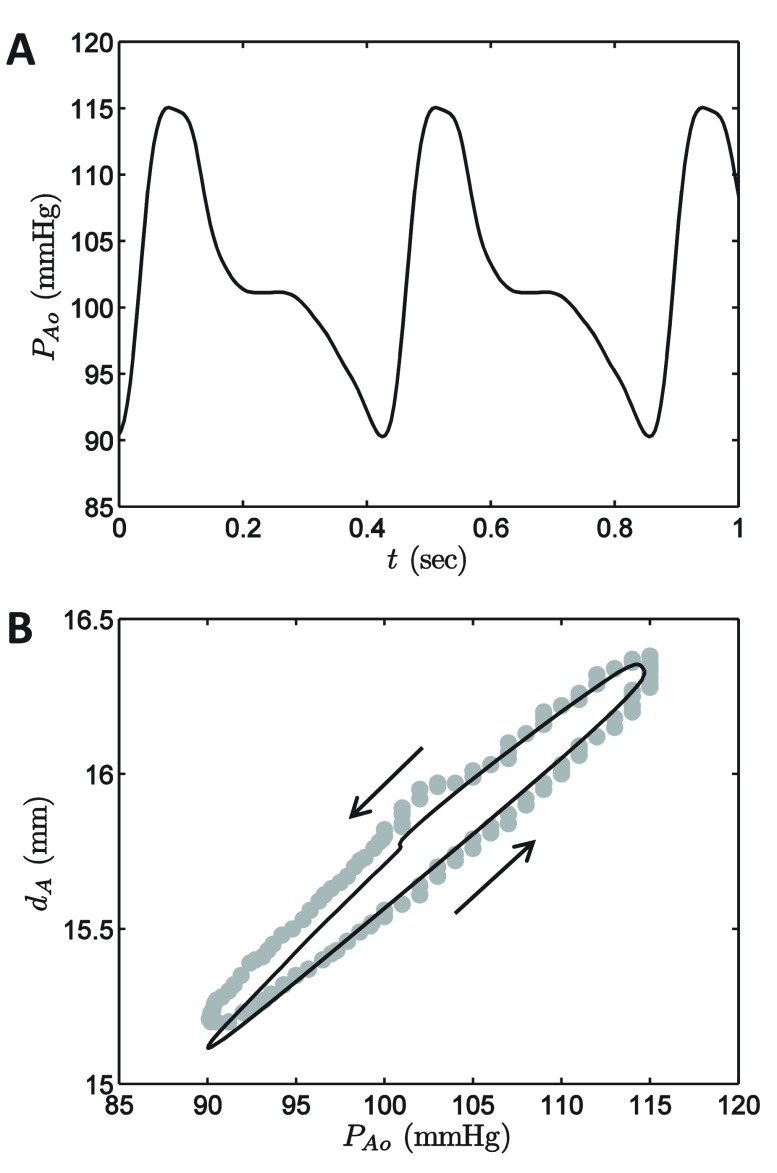
Simulated aortic mechanics. **A**. The aortic pressure time course obtained from Coleridge *et al.* ^11^ is used as the input to the aortic mechanics model module, Equation (4). **B**. The model-predicted relationship between aortic pressure and diameter is compared to the data reported by Coleridge *et al.* ^11^. Model simulations are plotted as a solid black cure; data are plotted as shaded circles.

The parameters of the large-artery mechanics model component are identified based on measurement of aortic diameter and pressure in dogs reported by Coleridge *et al.* ^12^. Figure 1 plots the measured aortic pressure wave in the upper panel and vessel diameter as a function of pressure in the lower. The experiment of Coleridge *et al.* is simulated using the governing equations

$$\begin{array}{l} \tau_{cAo}\frac{dV_{sAo}}{dt}=V_{sAo}^{\infty }-V_{sAo}\\ \frac{dV_{Ao}}{dt}=C_{Ao}\frac{dP_{Ao}}{dt}+\frac{dV_{sAo}}{dt}\\ \frac{d\varepsilon }{dt}=\frac{1}{2{\left(V_{Ao}V_{0}\right)}^{1/2}}\frac{dV_{Ao}}{dt},\;\;\;\;\;(4) \end{array}$$

where the measured aortic pressure waveform ( Figure 1A) is used to numerically approximate *dP _A0_*( *t*)/ *dt* in integrating Equation (4).

The parameters *γ _Ao_*, *C _Ao_*, and *τ _cA_*, were adjusted to match the data in Figure 1B; the values of *V*_0_ and *d*_0_ were set arbitrarily by assuming a cylindrical vessel of length 30 mm. All parameter values are listed in Table 1.

**Table 1.** Model parameters.

*Aorta/large-artery mechanics*		*Sensitivity*
*V*_0_ = 0.6875 ml	Aortic mechanics, Equation (1)	*
*d*_0_ = 12 mm	Aortic mechanics, Equation (1)	*
*C _Ao_* = 0.007 ml mmHg ^-1^	Aorta acute capacitance, Equation (2)	191
*γ _Ao_* = 0.40	Aorta creep parameter, Equation (3)	79.6
*τ _cAo_* = 0.12 sec	Aorta creep time constant, Equation (3)	0.20
*Kinetics of baroreflex afferent firing*		
*τ _s_* = 251.5 sec	Baroreceptor parameter, Equation (5)	0.16
*a* = 0.0651 sec ^-1^	Baroreceptor activation rate, Equation (7)	0.21
*b* = 0.2004 sec ^-1^	Baroreceptor deactivation rate, Equation (7)	0.35
*δ*_0_ = 0.4965	Baroreceptor saturation constant, Equation (7)	0.74
*f*_0_ = 299.8 sec ^-1^	Baroreceptor gain parameter, Equation (6)	2.55
*Mechanics of the heart and circulation*		
*E*_max_ = 8 mmHg ml ^-1^	Varying elastance heart model, Equation (10)	*
*E*_min_ = 0.25 mmHg ml ^-1^	Varying elastance heart model, Equation (10)	*
*T _M_* = 0.3	Varying elastance heart model, Equation (10)	*
*T _R_* = 0.15	Varying elastance heart model, Equation (10)	*
*H*_0_ = 75 beat min ^-1^	Varying elastance heart model, Equation (11)	*
*H*_1_ = 100 beat min ^-1^	Varying elastance heart model, Equation (11)	*
*R _out_* = 1×10 ^-4^ mmHg min ml ^-1^	Aortic valve resistance, Equation (12)	*
*R _Ao_* = 3×10 ^-4^ mmHg min ml ^-1^	Aortic resistance, Equation (12)	*
*R _A_*_0_ = 0.01160 mmHg min ml ^-1^	Large-artery resistance, Equation (12) and Equation (13)	*
*R _V_* = 3.359×10 ^-4^ mmHg min ml ^-1^	Downstream (venous) resistance, Equation (12)	*
*C _A_*_0_ = 0.8185 ml mmHg ^-1^	Large-artery compliance, Equation (13)	*
*C _V_*_0_ = 329.9 ml mmHg ^-1^	Downstream (venous) resistance, Equation (13)	*
*V _V_*_01_ = 625.1 ml	Unstressed volume of cardiovascular system, Equation (17)	1.82
*γ _V_* = 0.40	Venous creep parameter, Equation (17)	*
*τ _cV_* = 120 sec	Venous creep time constant, Equation (17)	*
*α*_1_ = 0.319	Arterial and venous compliance parameter, Equation (13)	0.36
*α*_2_ = 14.18	Arterial resistance parameter, Equation (13)	0.05
*α*_3_ = 1.036	Arterial and venous compliance parameter, Equation (13)	0.40
*α*_4_ = 2.969	Arterial resistance parameter, Equation (13)	0.60
*F*_0_ = 1125 ml min ^-1^	Autoregulation parameter, Equation (14)	5.96
*F*_1_ = 521.3 ml min ^-1^	Autoregulation parameter, Equation (14)	0.82
*τ _AR_* = 6.77 min	Autoregulation time constant, Equation (14)	0.20
*τ _F_* = 15 sec	Arbitrary time constant, Equation (15)	*
*Autonomic system*		
*f _SN_* = 2.76 sec ^-1^	Baroreflex arc parameter, Equation (18)	5.5
*Renin-angiotensin system*		
*τ _R_* = 12.61 min	Time constant for renin production, Equation (19)	0.30
*τ _A2_* = 1.117 min	Time constant for angiotensin II production, Equation (20)	0.30
*τ _P_* = 15 sec	Time constant for mean pressure calculation, Equation (19)	*
*P*_1_ = 20.12 mmHg	Steady-state renin-angiotensin system tone, Equation (19)	0.17
*P _2_* = 24.98 mmHg	Steady-state renin-angiotensin system tone, Equation (19)	0.37
*g* = 245.9 mmHg	Steady-state renin-angiotensin system tone, Equation (19)	1.05
*Neurohumoral control of pressure-diuresis/natriuresis*		
*k*_1_ = 0.125 ml sec ^-1^ mmHg ^-1^	Slope of acute pressure-diuresis relationship, Equation (21)	*
*P _s,_*_max_ = 126.4 mmHg	Long-term pressure-diuresis relationship, Equation (22)	*
*P _s,_*_min_ = 9.779 mmHg	Long-term pressure-diuresis relationship, Equation (22)	*
*ϕ*_0_ = 0.1928	Long-term pressure-diuresis relationship, Equation (22)	*
*ϕ*_1_ = 0.4813	Long-term pressure-diuresis relationship, Equation (22)	*
*τ _k_* = 10 min	Time constant for long-term pressure-diuresis, Equation (22)	0.09

* parameter not identified based on fitting time-course data; see text for details

### Model Components 2: Kinetics of baroreflex afferent firing

The baroreceptor afferent firing rate is assumed to be governed by the rate of change of strain in the vessel wall. The model invokes a moving average strain value $\bar{\varepsilon }$( *t*), which is computed

$$\tau_{s}\frac{d\bar{\varepsilon }}{dt}=\varepsilon -\bar{\varepsilon }.\;\;\;\;\;(5)$$

The time constant *τ _s_* is an adjustable parameter. The baroreceptor firing rate is assumed proportional to *δ _ε_* = max( *ε*– $\bar{\varepsilon }$,0) via the saturable relationship

$$f_{BR}(t)=f_{0}s(t)\frac{\delta_{\varepsilon }(t)}{\delta_{\varepsilon }(t)+\delta_{0}},\;\;\;\;\;(6)$$

where *s*( *t*) represents the fraction of baroreceptor afferents that are in an active/permissible state, and *δ*_0_ and *f*_0_ are additional adjustable parameters. Equation (6) is a static nonlinearity ^13^ that enforces a saturating response. It is assumed that the baroreceptors within the population transition from an active to inactive state at a rate proportional to the firing rate, and transition to the active state at a constant rate:

$$\frac{ds}{dt}=a\left(1-s\right)-bs\frac{\delta_{\varepsilon }}{\delta_{\varepsilon }+\delta_{0}}.\;\;\;\;\;(7)$$

The adjustable parameters in the baroreflex afferent model ( *τ _s_*, *δ*_0_, *f*_0_, *a*, and *b*) are identified based on measurements following step changes in non-pulsatile carotid sinuspressure ^14^ and ramps in *in vivo* pulsatile aortic pressure ^12^.

**Figure 2.**
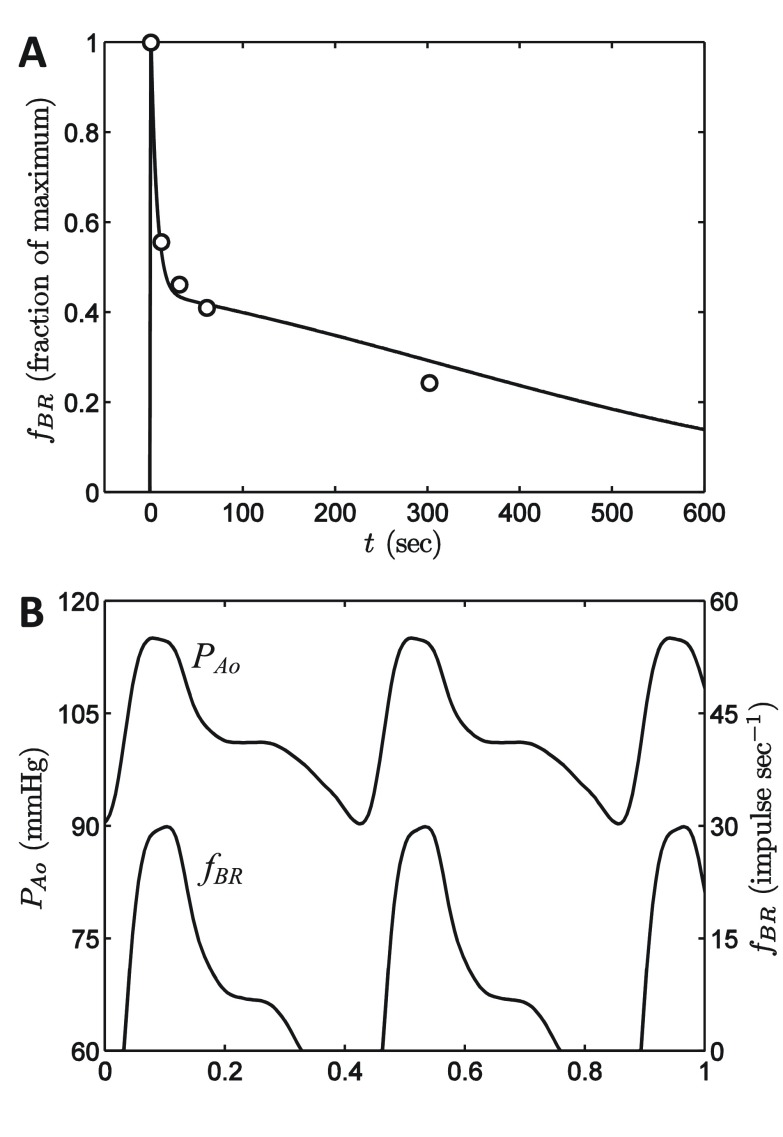
Baroreflex model. **A**. The response of the baroreflex model to a step increase in pressure is compared to data from Chapleau *et al.* ^13^. The model simulations are based on Equation (4)– Equation (7), as described in the text. **B**. Simulated baroreflex output based on input aortic pressure wave obtained from Coleridge *et al.* ^11^.

The data in Figure 2A, obtained from Chapleau *et al.* ^14^, correspond to the multi-fiber response of the carotid sinus baroreceptors following a step change in pressure from 40 mmHg to 93 mmHg. To simulate this experiment, the pressure is assumed to follow a time course described by

$$P_{Ao}(t)=\left\{ \begin{array}{l} 40,t<-1\\ 40+53\cdot \left(t+1\right),t\geq -1,\\ 93,t\geq 0 \end{array}\right.\;\;\;\;\;(8)$$

where pressure is expressed in units of mmHg and time in seconds. Thus it is assumed that pressure is increased by +53 mmHg over an interval from -1 to 0 seconds. Since the overall model does not differentiate between aortic and carotid baroreflex signals, data from the carotid pressure step are matched to the model of Equation (4)– Equation (7) in Figure 2A. Following the step pressure increase the afferent firing rate *f _BR_* rapidly increases to a maximum value before decaying over a time scale of several hundred seconds. This decay is determined in the model by the combined action of mechanical relaxation governed by Equation (5) and inactivation governed by Equation (7).

Figure 2B plots model-predicted baroreflex firing rate elicited by the normal aortic pressure waveform of Coleridge *et al.* ^12^. The model produces the characteristic bursting pattern where peaks in baroreceptor afferent firing occur in systole, with the firing rate dropping to zero in diastole.

**Figure 3.**
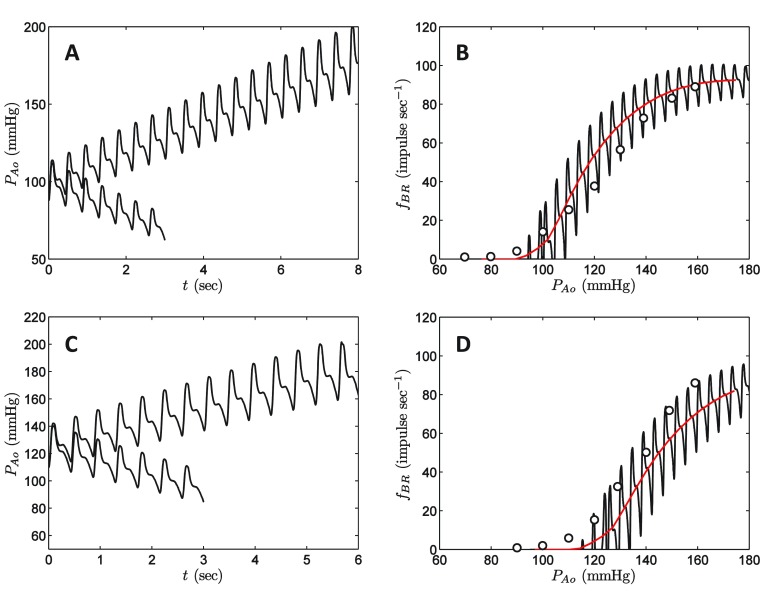
Baroreflex response to *in vivo* pressure ramps. **A**. Applied pressure transients for increasing and decreasing pressure starting at a baseline mean pressure of 100 mmHg. Pressure is increased/decreased so that the mean pressure changes at the rate of ±10 mmHg sec ^-1^. **B**. Simulated baroreceptor firing response to pressure ramps from **A** is compared to data from Coleridge *et al.* ^11^. **C**. Applied pressure transients for increasing and decreasing pressure starting at a baseline mean pressure of 125 mmHg. **D**. Simulated baroreceptor firing response to pressure ramps from **C** is compared to data from Coleridge *et al.* ^11^. See text for details on simulation protocol. In **B** and **D**, experimental data are plotted as circles; black line represents the simulation prediction; red line represents simulation predictions averaged over each heart beat.

Responses to ramps of pressure *in vivo *are compared to data of Coleridge *et al.* in Figure 3. Coleridge *et al.* adjusted *in vivo* pressure in the aortic sinus by placing hydraulic occlusion at various positions along the aorta. Model simulations are based on applying a constant increase/decrease to measured *P _Ao_*( *t*), resulting in the pressure time series plotted in Figure 3A and 3C. It is assumed that mean pressure is adjusted at a rate of ±10 mmHg sec ^-1^, corresponding to the experimental measurements. The top panel in the figure corresponds to simulations and data associated with the baseline state, where initial mean pressure is 100 mmHg. Data on afferent firing rate for this experiment are plotted as open circles in Figure 3B. The solid line in Figure 3B represent simulated *f _BR_*( *t*) versus *P _Ao_*( *t*). The red line represents *f _BR_*( *t*) and *P _Ao_*( *t*) averaged over each beat. The lower panel in Figure 3 corresponds to a hypertensive state where the aortic pressure was held at an elevated level with mean of 125 mmHg for 20 minutes prior to the pressure ramp experiment. Thus the system has had 20 minutes to adapt or “reset” to a new mean pressure, resulting in a right shift of the baroreflex response *f _BR_* versus *P _Ao_*.

The values of the parameters *τ _s_*, *δ*_0_, *f*_0_, *a*, and *b* were adjusted to match the data in Figure 2A and Figure 3.

### Model Components 3: Mechanics of the heart and circulation

**Figure 4.**
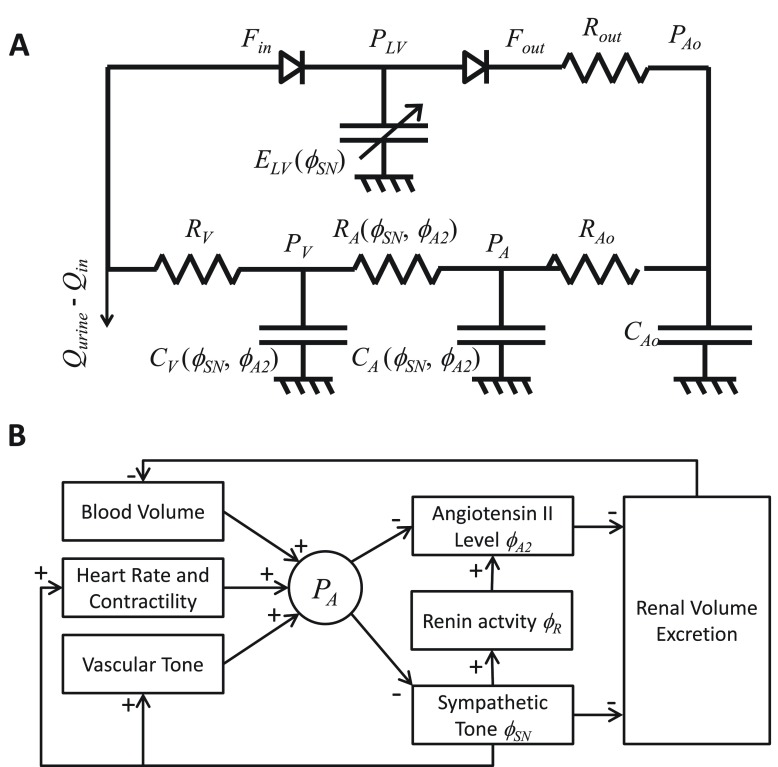
Overview of the whole-body model. **A**. Diagram of cardiovascular circuit model representing the systemic circulation. Flows entering and exciting the heart are denoted *F _in_* and *F _out_*; pressure in the aorta and arterial and venous capacitors are denoted *P _Ao_*, *P _A_* and *P _V_*; left-ventricular pressure is denoted *P _LV_*. **B**. Diagram of control systems captured by the model.

The circulation is modeled as a closed-loop lumped-parameter circuit illustrated in Figure 4A, which ignores the pulmonary circulation and treats the heart as time-varying elastance representing the left ventricle ^15^. The left-ventricular pressure is described by

$$P_{LV}(t)=E_{LV}(t)\cdot V_{LV}(t),\;\;\;\;\;(9)$$

where *E _LV_*( *t*) is the left-ventricular elastance and *V _LV_*( *t*) is the volume of blood in the ventricle. The elastance is simulated using a smooth function that increases in systole and decreases as the heart relaxes:

$$E_{LV}(\theta )=\left\{ \begin{array}{l} \frac{\left((0.75+\phi_{SN})E_{\max}-E_{\min}\right)}{2}\left[1-\cos\left(\frac{\pi \theta }{T_{M}}\right)\right]+E_{\min}\;\;\;\;\;\;0\leq \theta \leq T_{M}\\ \frac{\left((0.75+\phi_{SN})E_{\max}-E_{\min}\right)}{2}\left[\cos\left(\frac{\pi \left(\theta -T_{M}\right)}{T_{R}}\right)+1\right]+E_{\min}\;\;T_{M}\leq \theta \leq T_{M}+T_{R},\;\;\;\;\;(10)\\ E_{\min\;\;\;\;\;\;\;\;\;\;\;\;\;\;\;\;\;\;\;\;\;\;\;\;\;\;\;\;\;\;\;\;\;\;\;\;\;\;\;\;\;\;\;\;\;\;\;\;\;\;\;\;\;\;\;\;\;\;\;\;\;\;\;\;\;\;\;\;\;\;}T_{M}+T_{R}\leq \theta \leq 1 \end{array}\right.$$

where *θ* Є (0,1) is the fraction of a heart beat that has elapsed at a given time. The parameters *E*_max_ and *E*_min_ represent minimum effective elastance in the ventricle during systole and diastole. The factor (0.75 + *ϕ _SN_*) multiplying *E*_max_ accounts for the effect of sympathetic tone on contractility, where *ϕ _SN_* ( *t*) Є (0,1) is a model variable (see below) representing the sympathetic drive. Under baseline conditions *ϕ _SN_* ≈ 0.25 and therefore, under maximal sympathetic stimulation, cardiac contractility is approximately 175% of baseline.

The variable *θ* is simulated via

$$\frac{d\theta }{dt}=H,\;\;\;\;\;(11)$$

where *H* = *H*_0_ + *H*_1_ ( *ϕ _SN_* -0.25) is the heart rate, and *θ*( *t*) is reset to 0 each time the value reaches 1. The parameters *H*_0_ and *H*_1_ are set to give a baseline heart rate of 75 beats min ^-1^ and a heart rate of 150 beats min ^-1^ under maximal sympathetic tone.

The circuit model is simulated based on equations for the state variables *θ*( *t*), *V _LV_*( *t*), *V _Ao_*( *t*), *V _A_*( *t*), *V _V_*( *t*), *V _sA_*( *t*), and *V _sV_*( *t*). The governing equations for the six volume variables are

$$\begin{array}{l} \frac{dV_{LV}}{dt}=\max\left(0,\frac{P_{v}-P_{LV}}{R_{V}}\right)-\max\left(0,\frac{P_{LV}-P_{Ao}}{R_{out}}\right),\\ \frac{dV_{Ao}}{dt}=\max\left(0,\frac{P_{LV}-P_{Ao}}{R_{out}}\right)-\frac{P_{Ao}-P_{A}}{R_{Ao}},\\ \frac{dV_{A}}{dt}=\frac{P_{Ao}-P_{A}}{R_{Ao}}-\frac{P_{A}-P_{V}}{r_{AR}R_{A}},\\ \frac{dV_{V}}{dt}=\frac{P_{A}-P_{V}}{r_{AR}R_{A}}-\max\left(0,\frac{P_{V}-P_{LV})}{R_{V}}\right)+Q_{input}-Q_{urine\;},\\ \frac{dV_{SAo}}{dt}=\left(V_{sAo}^{\infty }-V_{sAo}\right)/\tau_{cAo\;},\\ \frac{dVsv}{dt}=\left(V_{sV}^{\infty }-V_{sV}\right)/\tau_{cV},\;\;\;\;\;\;\;\;\;\;(12) \end{array}$$

where *P _LV_*( *t*) is determined by Equation (9).

The flows *Q _in_* and *Q _urine_* represent the rates of volume uptake/infusion and urine production, described below.

The max(0,∙) terms in Equation (12) account for the valves, which permit flow only in the direction indicated in Figure 4A.

The neurohumoral control mechanisms captured by the model are diagrammed in Figure 4B. The *R _Ao_* and *R _V_* resistances are set to constant values, while other resistances and capacitances vary with sympathetic tone and angiotensin II level. Specifically, *C _A_*( *t*), *C _V_*( *t*), and *R _A_*( *t*) are determined by

$$\begin{array}{l} C_{A}(t)=\frac{C_{A0}}{\left(1+\alpha_{1}\phi_{SN}\left(t\right)\right)\left(1+\alpha_{3}\phi_{A2}\left(t\right)\right)}\\ C_{V}(t)=\frac{C_{V0}}{\left(1+\alpha_{1}\phi_{SN}(t))(1+\alpha_{3}\phi_{A2}(t)\right)}\\ R_{A}(t)=R_{A0}(1+\alpha_{2}\phi_{SN}(t))(1+\alpha_{4}\phi_{A2}(t)),\;\;\;\;\;(13) \end{array}$$

where *C _A_*_0_, *C _V_*_0_, and *R _A_*_0_ are constants, and *α*_1_, *α*_2_, *α*_3_, and *α*_4_ are constants that determine the magnitude of the effects of vasoconstriction via sympathetic tone and angiotensin II. The variable *ϕ _A2_* represents the plasma angiotensin II activity. The governing equations for *ϕ _A2_* are described below.

The *R _A_* resistance is further governed by a whole-body autoregulation phenomenon, as incorporated into the Guyton-Coleman models ^6, 8, 9^. The function *r _AR_*( *t*) Є (0,1) accounts for autoregulatory effects on systemic arterial conductivity based on

$$\begin{array}{l} \tau_{AR}\frac{r_{AR}}{dt}=r_{AR}^{\infty }-r_{AR}\\ r_{AR}^{\infty }=\frac{1}{2}\left(1+\tanh\left(\frac{\bar{F}-F_{0}}{F_{1}}\right)\right)\;\;\;\;\;(14) \end{array}$$

where $\bar{F}$( *t*) is a variable that averages the mean cardiac output with a moving average defined by a first-order process:

$$\tau_{F}\frac{d\bar{F}}{dt}=\frac{P_{A}-P_{V}}{r_{AR}R_{A}}-\bar{F}\cdot \;\;\;\;\;(15)$$

The parameters *F*_0_ and *F*_1_ determine the autoregulatory response of resistance to changes in flow. The pressures are computed from the relationships

$$\begin{array}{l} P_{Ao}=\left(V_{Ao}-V_{sAo}\right)/C_{Ao}\\ P_{A}=V_{A}/C_{A}\\ P_{V}=\left(V_{V}-V_{sV}-V_{V01}\right)/C_{V},\;\;\;\;\;(16) \end{array}$$

in which venous compliance is simulated using a linear formulation of stress relaxation similar to that used for the aorta. Specifically, venous stress relaxation kinetics are governed by *τ _cV_*( *V _sV_/dt*) = *V _sV_^∞^ – V _sV_*, where

$$V_{sV}^{\infty }=\left(1-\frac{C_{V}}{C_{\infty }}\right)\left(V_{V}-V_{V01}\right)=\gamma_{V}\left(V_{V}-V_{V01}\right).\;\;\;\;\;(17)$$

The constant *V _V_*_01_ in Equation (17) represents an unstressed volume for the overall cardiovascular system.

**Figure 5.**
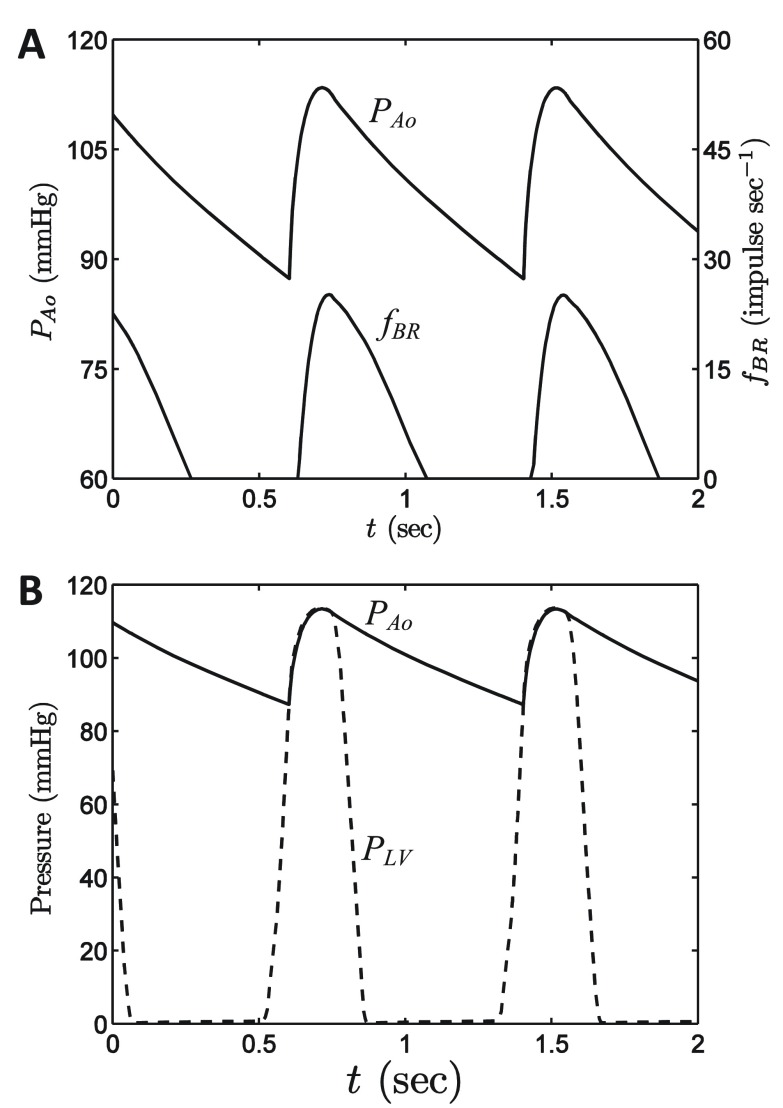
Baseline model operation. **A**. Model-predicted aortic pressure and baroreflex firing rate, obtained with *Q _in_* = 0.5835 ml min ^-1^. This simulation represents a period steady-state of the model, in which *Q _in_* = *Q _out_* and average pressure is 100 mmHg. **B**. Model-predicted aortic and left-ventricular pressures are plotted for the baseline period steady state.

There are a total of 23 parameters associated with this component of the model. Assignment of the values listed in Table 1 was guided by a variety of data sets and previous computational models. The values of *E*_max_ and *E*_max_ were obtained by scaling the model of ^16^ to provide reasonable pressure under baseline conditions at ventricular volumes appropriate for dog. The cardiac cycle timing parameters *T _M_* and *T _R_* were set to the values used by Beard ^16^. Heart rate parameters *H*_0_ and *H*_1_ were determined as described above. The baseline resistance and compliance parameters *R _out_*, *R _Ao_*, *R _A_*_0_, *R _V_*, *C _A_*_0_, *C _V_*_0_, and *V _V_*_01_ were chosen so that under baseline conditions ( Figure 5), the mean pressure is 100 mmHg, the diastolic and systolic pressures are 85 and 115 mmHg, and the ejection fraction is 0.58. (The values for these seven parameters do not represent a unique set that gives these outputs under baseline conditions). The parameters *γ _V_* and *τ _cV_* were set to match measurement of stress relaxation in the canine jugular ^17^.

The remaining seven parameters in the cardiac and circulation model component ( *α*_1_, *α*_2_, *α*_3_, *α*_4_, *F*_0_, *F*_1_, and *τ _AR_*) were identified by simulating the responses of the system to volume infusion and hemorrhage, as detailed below in Results.

### Model Components 4: Autonomic system

The whole-body sympathetic tone is represented in the model by the variable *ϕ _SN_*( *t*) Є (0,1) and is determined by the baroreflex arc:

$$\frac{d\phi_{SN}}{dt}=f_{SN}\left(1-\phi_{SN}\right)-f_{BR}\phi_{SN}.\;\;\;\;\;(18)$$

Thus, in the absence of baroreflex firing, the sympathetic tone will approach the maximum value of 1. The constant parameter *f _SN_* is set so that under baseline conditions *ϕ _SN_*( *t*) = 0.25. Thus, following a rapid severe drop in pressure *ϕ _SN_*( *t*) will approach a value that is four times the baseline value.

### Model Components 5: Renin-angiotensin system

The state of the renin-angiotensin system is captured by two variables: *ϕ _R_*( *t*) Є (0,1) and *ϕ _A2_*( *t*) Є (0,1), which represents plasma renin and angiotensin II activity governed by the combined action of sympathetic tone and pressure on renin release.

The plasma renin variable *ϕ _R_*( *t*) is governed by

$$\begin{array}{l} \tau_{R}\frac{d\phi_{R}}{dt}=\phi_{R}^{\infty }-\phi_{R}\\ \tau_{P}\frac{d\bar{P}}{dt}=P_{A}-\bar{P}\\ \phi_{R}^{\infty }=\frac{1}{2}\left(1-\tanh\left(\frac{\bar{P}-g\phi_{SN}-P_{1}}{P_{2}}\right)\right),\;\;\;\;\;(19) \end{array}$$

where *ϕ _R_^∞^* decreases with increasing time-averaged arterial pressure $\bar{P}$. The parameters *P*_1_ and *P*_2_ determine the shape of the steady state relationship between renin production and pressure. This formulation assumes that the relationship between steady-state *ϕ _R_* and pressure $\bar{P}$ is shifted by the sympathetic tone. Based on observations showing that plasma renin activity and angiotensin II levels are nearly perfectly linearly related *in vivo* ^18^, we assumed that *ϕ _A2_*( *t*) follows *ϕ _R_*( *t*) according to

$$\tau_{A2}\frac{d\phi_{A2}}{dt}=\phi_{R}-\phi_{A2}.\;\;\;\;\;(20)$$

These expressions simplify the known (and unknown) mechanisms governing renin production into a simple phenomenological relationship between pressure, sympathetic tone, and the kinetics of the renin-angiotensin system. Here the kinetics of the renin-angiotensin system are treated with much less detail than other models, which account for whole-body pharmacokinetics ^19^ and saturable enzyme kinetics ^20^. The five parameters invoked in this model component ( *τ _R_*, *τ _A2_*, *g*, *P*_1_, and *P*_2_) are identified based on comparing simulations to measurements of pressure, heart rate, and plasma renin activity in rabbit during graded hemorrhage, as detailed below in Results. (The simulation parameter *τ _P_* was arbitrarily set at 15 seconds).

### Model Components 6: Neurohumoral control of pressure-diuresis/natriuresis

Regulation of body-fluid volume is assumed governed by a linear pressure-diuresis relationship:

$$Q_{urine}=\left\{ \begin{array}{l} 0,\;\;\;\;\;\;\;\;\;\;\;\;\;\;\;\;\;\;\;\bar{P}<P_{s}\\ \min(k_{1}\cdot \left(\bar{P}-P_{s}\right),10{\text{ml min}}^{-1}),\;\;\;\bar{P}\geq P_{s}\;\;\;\;\;\;(21) \end{array}\right.$$

where *Q _urine_* is rate of volume output via the kidneys and *k*_1_ is the slope of the relationship between *Q _urine_* and pressure. The model variable *P _s_*( *t*) is the variable offset of the pressure-diuresis relationship, which is controlled by sympathetic tone and angiotensin II level:

$$\begin{array}{l} P_{s}^{\infty }=P_{s,\min}+\frac{P_{s,\max}-P_{s,\min}}{2}\left(1+\tanh\left(\frac{\phi_{SN}+\phi_{A2}-\phi_{0}}{\phi_{1}}\right)\right)\\ \tau_{k}\frac{dP_{s}}{dt}=P_{s}^{\infty }-P_{s}.\;\;\;\;\;\;\;\;\;\;\;\;\;\;\;\;\;\;\;\;\;\;\;\;\;(22) \end{array}$$

The constant parameters *P _s,_*_min_, *P _s,_*_max_, *ϕ*_0_, and *ϕ*_1_ determine how the acute pressure-diuresis relationship shifts in response to changes in the tone variables *ϕ _SN_* and *ϕ _A2_*. Thus it is assumed that *ϕ _SN_* and *ϕ _A2_* have equal and additive effects on renal function. Equation (22) assumes that changes in *P _s_* governed by sympathetic tone and the renin-angiotensin system occur with a time constant *τ _k_*. Furthermore, the maximal rate of urine production is set to 10 ml min ^-1^.

The parameters *k*_1_, *P _s,_*_max_, *P _s,_*_min_, *ϕ*_0_, and *ϕ*_1_, were set by matching model predictions to data on the pressure diuresis relationship under physiological conditions, with angiotensin II infusion, and with administration of an angiotensin converting enzyme (ACE) inhibitor. To compare predictions of Equation (21) and Equation (22) to renal output measurements under ACE inhibition, we assumed that for the chronic measurements, sympathetic tone was maintained at its baseline value and *ϕ _A2_* = 0. Fitting data from Hall *et al.* ^21^, it is estimated that *P _s_* ^∞^ ( *ϕ _SN_* = 0.25, *ϕ _A2_* = 0) = 75 mmHg. Similarly, with angiotensin II infusion, we assumed that sympathetic tone was maintained at its baseline value and *ϕ _A2_* = 1. The data of Hall *et al.* yield an estimate of *P _s_^∞^* ( *ϕ _SN_* = 0.25, *ϕ _A2_* = 1.0) = 125 mmHg for these conditions. Finally, under baseline conditions, with $\bar{P}$ = 100 mmHg, urine output was estimated to be *Q _urine_* = 0.5835 ml min ^-1^, based on volume infusion experiments described in the Results. With *k*_1_ = 0.125 ml sec ^-1^ mmHg ^-1^ (see Results), and using the baseline *ϕ _A2_* value of 0.1864 (which is determined from blood withdrawal experiments; see Results), it follows that *P _s_^∞^* ( *ϕ _SN_* = 0.25, *ϕ _A2_* = 0.1864) = 95.3 mmHg. These estimates of *P _s_^∞^* at three different values of *ϕ _SN_* + *ϕ _A2_* were used to estimate the values of *P _s,_*_max_, *P _s,_*_min_, *ϕ*_0_, and *ϕ*_1_.

The time constant *τ _k_* was determined from data on the rate of urine production following infusion of blood leading to an acute increase in pressure and drop in *ϕ _SN_* and *ϕ _A2_*. Analysis of data from these experiments is detailed below in the Results together with a direct comparison between the data of Hall *et al.* ^21^ and model simulations.

## Results

### Results 1: Response to volume infusion

Guyton and colleagues conducted experiments in which a large amount of blood was infused into an anesthetized dog, resulting in a rapid increase in total blood volume of 45% compared to initial baseline value. The model of Equation (1)– Equation (22) was simulated and compared to data from this experiment to identify adjustable model parameters and to probe the predicted response of unmeasured model variables to this protocol.

**Figure 6.**
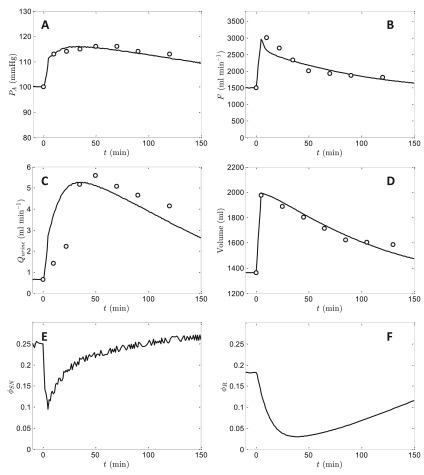
Simulation of arterial pressure, cardiac output, rate of urine formation, blood volume, *ϕ _SN_*, and *ϕ _R_* following infusion of 45% of initial baseline blood volume in a normal animal. Data on arterial pressure, cardiac output, rate of urine formation, and blood volume are obtained from Dobbs *et al.* ^18^, Guyton *et al.* ^1^, and Prather *et al.* ^19^. The initial steady state of the model was obtained based on a constant infusion of *Q _in_* = 0.5835 ml min ^-1^. For times 0 < *t* < 5 min, *Q _in_* was set to 0.5835 + 126.82 ml min ^-1^, to result in a total excess volume of 634 ml. The initial condition for the simulation was obtained by setting *Q _in_* = 0.5835 ml min ^-1^ and running the model to obtain the steady state.

Figure 6 shows the predicted effects of infusing 45% of initial baseline blood volume in a normal animal on arterial pressure, cardiac output, rate of urine formation, blood volume, sympathetic tone, and renin-angiotensin level together with experimental data obtained from Dobbs *et al.* ^22^, Guyton *et al.* ^1^, and Prather *et al.* ^23^ on the first four variables. (For these experiments Guyton *et al.* reported a baseline mean arterial pressure of approximately 115 mmHg. Since all other data sets analyzed here have a baseline pressure of 100 mmHg, 15 mmHg was subtracted from the data from these experiments to achieve consistency). The initial steady state of the model was obtained based on a constant infusion of *Q _in_* = 0.5835 ml min ^-1^, which matches the mean reported rate of volume output for the baseline data. For times 0 < *t* < 5 min, *Q _in_* was increased to 0.5835 + 126.82 ml min ^-1^, to result in a total excess volume of 634 ml. After the five-minute infusion, *Q _in_* was returned to the baseline value of 0.5835 ml min ^-1^. The model is able to effectively reproduce the trends in the experimentally measured variables, and predicts that *ϕ _SN_* and *ϕ _R_* drop to less than one half of their resting values following the volume infusion in response to the transient increase in pressure. These reductions in *ϕ _SN_* and *ϕ _R_* confer increases in vascular compliances helping to accommodate the substantial volume increase. As volume is removed from the system *ϕ _SN_* and *ϕ _R_* return toward the baseline values, with *ϕ _SN_* responding substantially faster than *ϕ _R_*.

The data plotted in Figure 6, along with data observations on graded hemorrhage, were used in identifying several adjustable parameters in the model. (See following section for details).

### Results 2: Response to hemorrhage

**Figure 7.**
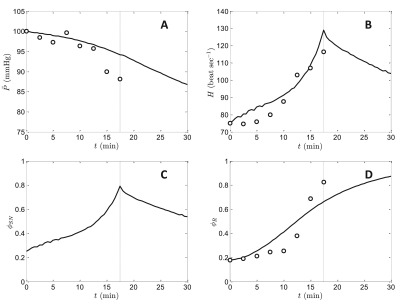
Simulation of system response to hemorrhage. The experimental and simulation protocol is to withdraw blood at a rate of 2% of initial total volume per minute, starting at time 0. The end of the withdrawal period, 17.5 minutes, is indicated in by dashed line in all plots. Model simulations are compared to data for mean pressure ( **A**), heart rate ( **B**), and plasma renin activity ( **D**). Panel **C** plots the model-predicted sympathetic tone during the protocol. The initial condition is the same baseline condition used for the simulations of Figure 6. Data from heart rate, pressure, and plasma renin activity following graded blood withdrawal are from Quail *et al.* ^18^ with pressure and heart rate scaled as described in the text.

Data from Quail *et al.* ^24^ on heart rate, pressure, and plasma renin activity following graded blood withdrawal in rabbits were used to identify parameters associated with the cardiovascular mechanics and the renin-angiotensin system. Figure 7 shows data measured in normal rabbits, where the heart rate measurements have been scaled to a baseline value of 75 beats min ^-1^, renin activity is scaled to maximum value of 1, and mean pressure is scaled to an initial baseline value of 100 mmHg. In the experiments, blood was withdrawn from the animals at a rate of 2% blood volume per minute. In model simulations, blood is withdrawn at the experimental rate starting at time 0 and with withdrawal stopped at time 17.5 minutes. For this experiment, the initial condition was identical to that used for the volume infusion experiments of Figure 6, and volume infusion and urine output were set to zero for the time course.

Model predictions of *H*, $\bar{P}$, and *ϕ _R_* compare favorably to experimental data, with mean arterial pressure dropping from the initial value of 100 mmHg to approximately 93 mmHg at the end of the blood withdrawal. The observed increase in heart rate is associated with an increase in *ϕ _SN_* from baseline level of 0.25 to 0.81, and an increase of *ϕ _R_* from baseline level of 0.186 to 0.668, at the termination of the withdrawal period. During this period, arterial blood pressure is protected from a more severe reduction in large part by an increase in vascular tone mediated by increases in sympathetic tone and angiotensin II level. Simulated cardiac output (not shown) also drops during blood withdrawal, reaching a minimum value of 690 ml min ^-1^ (approximately 46% of baseline flow) at 17.5 minutes.

The model is not able to capture the sharp, almost step-like, observed increase in renin that occurs following the 10-minute time point (corresponding to 20% of blood withdrawn). Specifically, the model predicts a more graded response, without the delay observed in the data. A better match to the data might be achieved by incorporating a delay into the governing equations, or by simulating the renin-angiotensin system using a higher-order system of differential equations than Equation (19) and Equation (20). The model predicts that *ϕ _SN_* and *H* peak at the end of the withdrawal period and partially recover toward baseline levels within a few minutes after the end of blood withdrawal. The renin-angiotensin tone is predicted to remain at an elevated level after the withdrawal. The partial drops in *ϕ _SN_* and *H* are associated with a graded decrease in pressure continuing over the entire simulated time course, with pressure reaching approximately 85 mmHg at 30 minutes. Pressure continues to drop after the end of the withdrawal period due in part to the action of the whole-body autoregulatory mechanism governed by Equation (16) and Equation (17). Via this mechanism, the drop in flow associated with hemorrhage causes a reduction in arterial resistance, resulting in a recovery in flow and a continuing drop in pressure.

The model predictions in Figure 6 and Figure 7 are most sensitive to the values of 13 parameters from the model components of circulatory mechanics, the renin-angiotensin system, and the kinetics of pressure-diuresis: *α*_1_, *α*_2_, *α*_3_, *α*_4_, *F*_0_, *F*_1_, *τ _AR_*, *τ _R_*, *τ _A2_*, *g*, *P*_1_, *P*_2_, and *t _K_*. The values of these parameters were adjusted to match the data plotted in Figure 6 and Figure 7.

### Results 3: Chronic renal function curves

**Figure 8.**
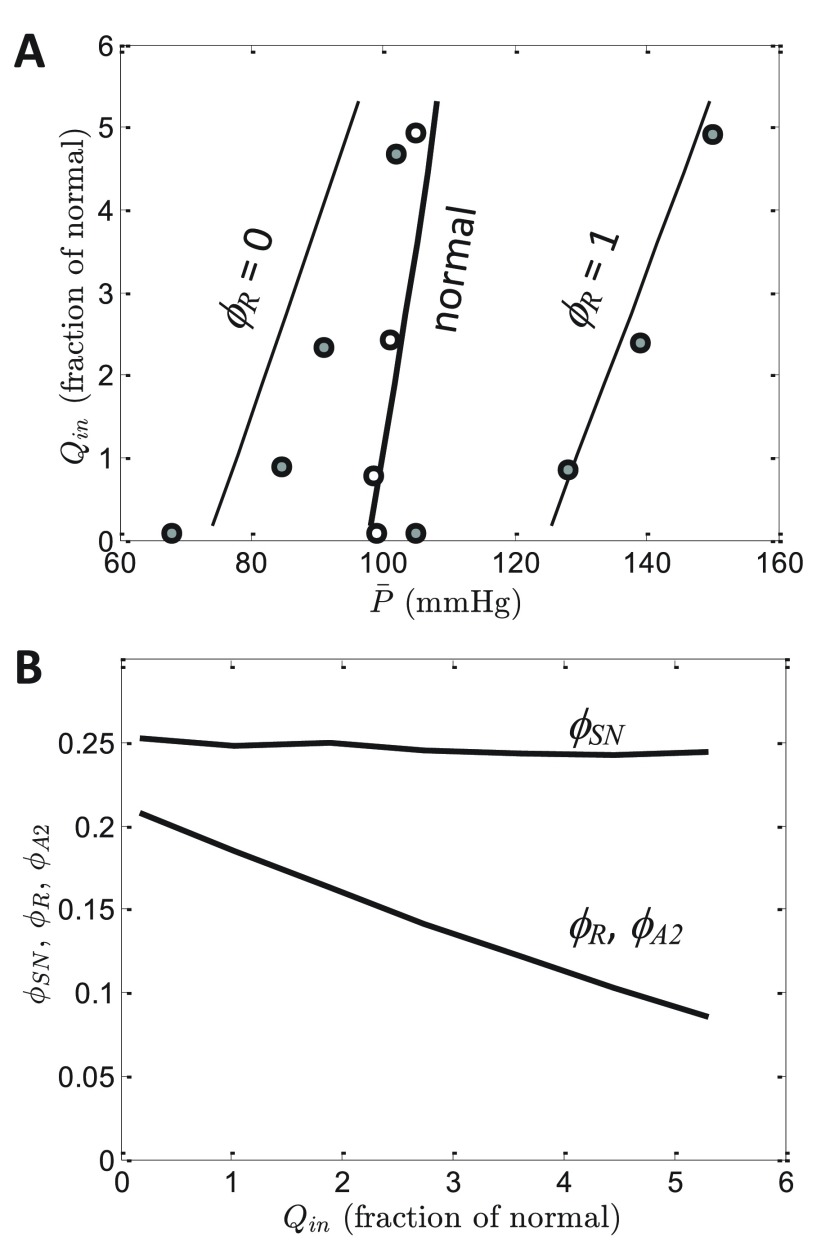
Renal function curves. **A**. Model predictions are compared to data on the steady-state relationship between mean arterial pressure and rate of volume infusion (equal to rate of urine output) for normal conditions, for angiotensin converting enzyme inhibition ( *ϕ _R_* = 0), and for angiotensin II infusion ( *ϕ _R_* = 1). Data are obtained from Hall *et al.* ^17^, in which net salt output is reported under these three conditions. Rate of urine volume production is assumed proportional to rate of sodium excretion, and normalized to the rate of urine production at baseline conditions ( $\bar{P}$ = 100 mmHg) for the normal case. **B**. Model prediction for steady-state renin and angiotensin II activities ( *ϕ _R_* and *ϕ _A2_*) and sympathetic tone ( *ϕ _SN_*) are plotted as functions of *Q _in_* for the normal case (without *ϕ _R_* clamped).

The steady-state relationship between arterial pressure and rate of urine output is obtained from model simulations by varying *Q _in_*, the rate of volume infusion, and attaining steady-state model predictions of steady state pressure where *Q _in_* = *Q _urine_*. Figure 8A plots the predicted relationship between rate of urine output and mean arterial pressure under three different conditions: (1) the normal physiological state; (2) *ϕ _R_* clamped at 0, representing complete block of angiotensin converting enzyme (ACE); and (3) *ϕ _R_* clamped at 1, representing infusion of saturating levels of angiotensin II. Note that the pressure-diuresis relationship is plotted in the traditional manner with arterial pressure on the abscissa and rate of volume excretion on the ordinate, even though in these simulations *Q _in_* is varied and steady-state $\bar{P}$ is computed as a function of *Q _in_*.

Model predictions are compared to data from Hall *et al.* ^21^ on steady-state pressure at different levels of salt/volume loading in normal dogs, dogs infused with ACE inhibitor, and dogs infused with angiotensin II. Model simulations effectively match experimental data, validating the assignment of parameter values described above under “Neurohumoral Control of Pressure-Diuresis/Natriuresis”.

Figure 8B illustrates model-predicted steady-state renin and angiotensin II activities ( *ϕ _R_* and *ϕ _A2_*) and sympathetic tone ( *ϕ _SN_*) as functions of *Q _in_* for the normal case. (Recall that from Equation (20) angiotensin II activity *ϕ _A2_* is equal to *ϕ _R_* in the steady state). As *Q _in_* is increased *ϕ _R_* and *ϕ _A2_* decrease to maintain pressure at nearly a constant level of the simulated range of volume/salt loading. The sympathetic tone on the other hand remains nearly constant around the baseline level of 0.25. Sympathetic tone remains constant because in the model the only determinant of *ϕ _SN_* is the baroreceptor afferent firing rate, which effectively adapts to the small changes in pressure that are associated with the simulated range of *Q _in_*. (Thus, the model does not capture suppression of sympathetic tone typically observed with chronic salt/volume loading).

### Results 4: Response to chronic baroreflex stimulation

**Figure 9.**
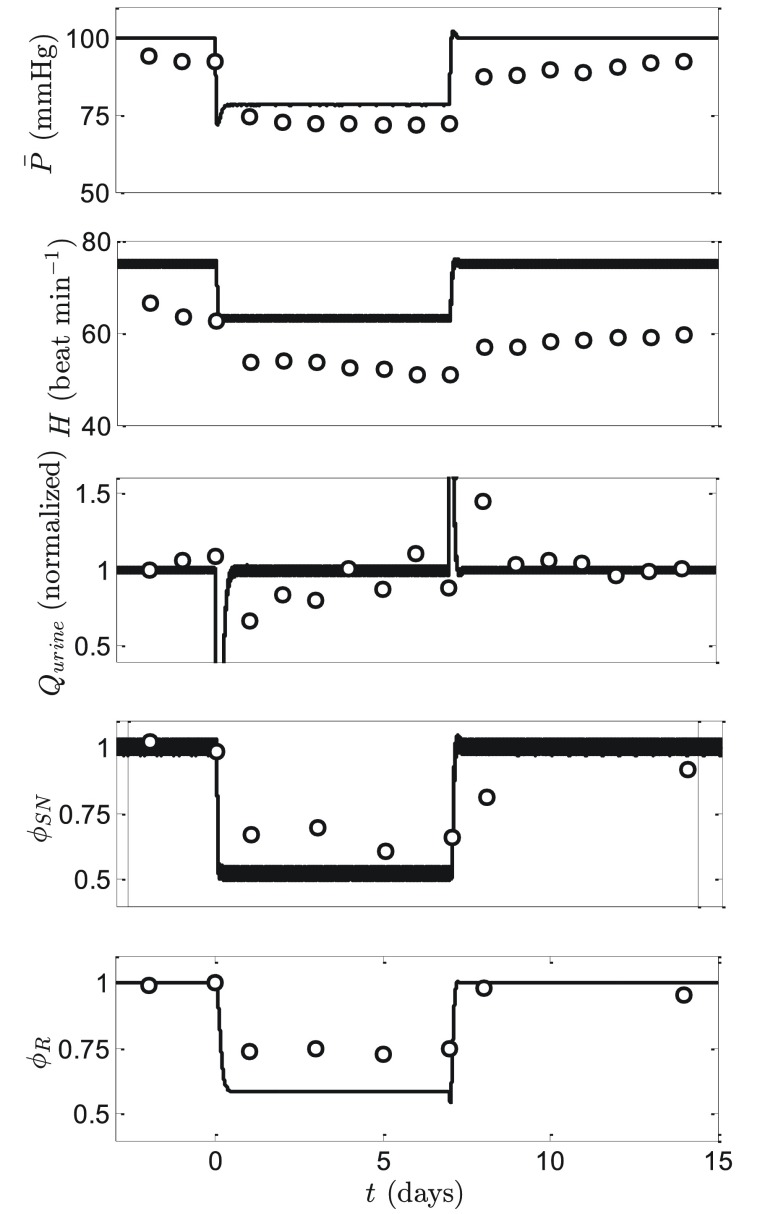
Response to chronic baroreflex stimulation. Electrical stimulation of the carotid baroreflex afferent nerve is simulated by modifying the normal model by replacing Equation (18) in the normal model with Equation (23) during the baroreflex stimulation period (for a 1-week period starting on day 0). Data on mean pressure, heart rate, urine output, plasma norepinephrine (plotted as *ϕ _SN_*), and plasma renin activity (plotted as *ϕ _RA_*) are obtained from Lohmeier *et al.* ^23, 24^.

Numerous studies have demonstrated that chronic stimulation of the carotid baroreceptor afferent nerve with implantable devices results in a prolonged decrease in mean arterial pressure ^25, 26^. Figure 9 illustrates a representative data set from Lohmeier *et al.* ^27, 28^ for which baroreflex stimulation was applied in dogs for a seven day period, followed by several days of recovery. During the stimulation period, mean arterial pressure drops by approximately 20%, renin activity by approximately 35%, and sympathetic tone (experimentally assayed indirectly by plasma norepinephrine) drops by approximately 55%.

To simulate this experiment, a constant was added to the model-predicted baroreceptor firing rate in the equation for sympathetic tone. Thus Equation (18) was replaced by

$$\frac{d\phi_{SN}}{dt}=f_{SN}\left(1-\phi_{SN}\right)-\left(f_{BR}+f_{stim}\right)\phi_{SN},\;\;\;\;\;(23)$$

where *f _stim_* is a parameter adjusted to match the experimental data. Using *f _stim_* = 6.5 sec ^-1^ yields predictions that effectively match the observations of Lohmeier *et al.* ^27, 28^ ( Figure 9). Indeed, given that only one parameter was adjusted, the comparison of model predictions to the five variables plotted in the figure represents a strong validation test for the model. In particular, the observed reduction in renin activity with baroreflex stimulation represents a phenomenon that has not been captured by previous modeling efforts ^26, 28^. Specifically, the model of Illiescu and Lohmeier predicts a substantial increase in renin production following initiation of stimulation followed by a return to the initial baseline level. In Illiescu-Lohmeier model, the transient increase in renin is a result of the drop in pressure causing an initial increase in renin production. The predicted initial increase is later offset by decreased sympathetic nerve activity ^24^. In our model the observed phenomenon of reduction in plasma renin activity emerges as a property of the integrated computational model: stimulation of the baroreflex and associated drop in sympathetic tone is predicted to result in a drop in renin production even with the long-term drop in mean pressure.

Thus our model prediction differs from that of the Illiescu-Lohmeier model in two critical ways. First, our model captures the observed long-term response of renin while the Illiescu-Lohmeier model does not. Second, our model predicts no increase in renin during the first day of baroreflex stimulation, while the Illiescu-Lohmeier model does. Illiescu and Lohmeier predict that an increase in renin “was not measured experimentally because initial blood sampling was not made until 24 h after initiating baroreflex activation” ^24^. In contrast, our model predicts that the increase in renin was not measured experimentally because it did not happen.

### Results 5: Exploration of the etiology of primary hypertension

Pettersen *et al.* ^29^ demonstrate that impairment of the baroreflex caused by stiffening of arterial vessels represents a viable hypothesis for the etiology of primary hypertension. Specifically, using a closed-loop cardiovascular mechanics model coupled to a detailed model of the baroreflex arc ^30^, Pettersen *et al.* ^29^ show that when the stiffness of the aorta is increased to represent changes in mechanical properties associated with ageing, the model based on their mechanogenic hypothesis predicts a substantial increase in mean arterial pressure with age. By assuming a constant blood volume for all age groups, the model explicitly did not account for the regulation of plasma volume and salt through the kidney and the renin-angiotensin system following from the hypothesized shift in the renal pressure-diuresis/natriuresis function curve. The rationale for not including adaptive mechanisms likely to partially ameliorate the effects of arterial stiffening on blood pressure was to test the explanatory sufficiency of the mechanogenic hypothesis by showing that it would predict a stronger relation between blood pressure increase and age than empirically observed. As a first step towards a complete merge of physiological renal function with the model developed by Pettersen *et al.* to expand the prediction space of the mechanogenic hypothesis, we studied how the current model responded to changes in the aortic compliance.

**Figure 10.**
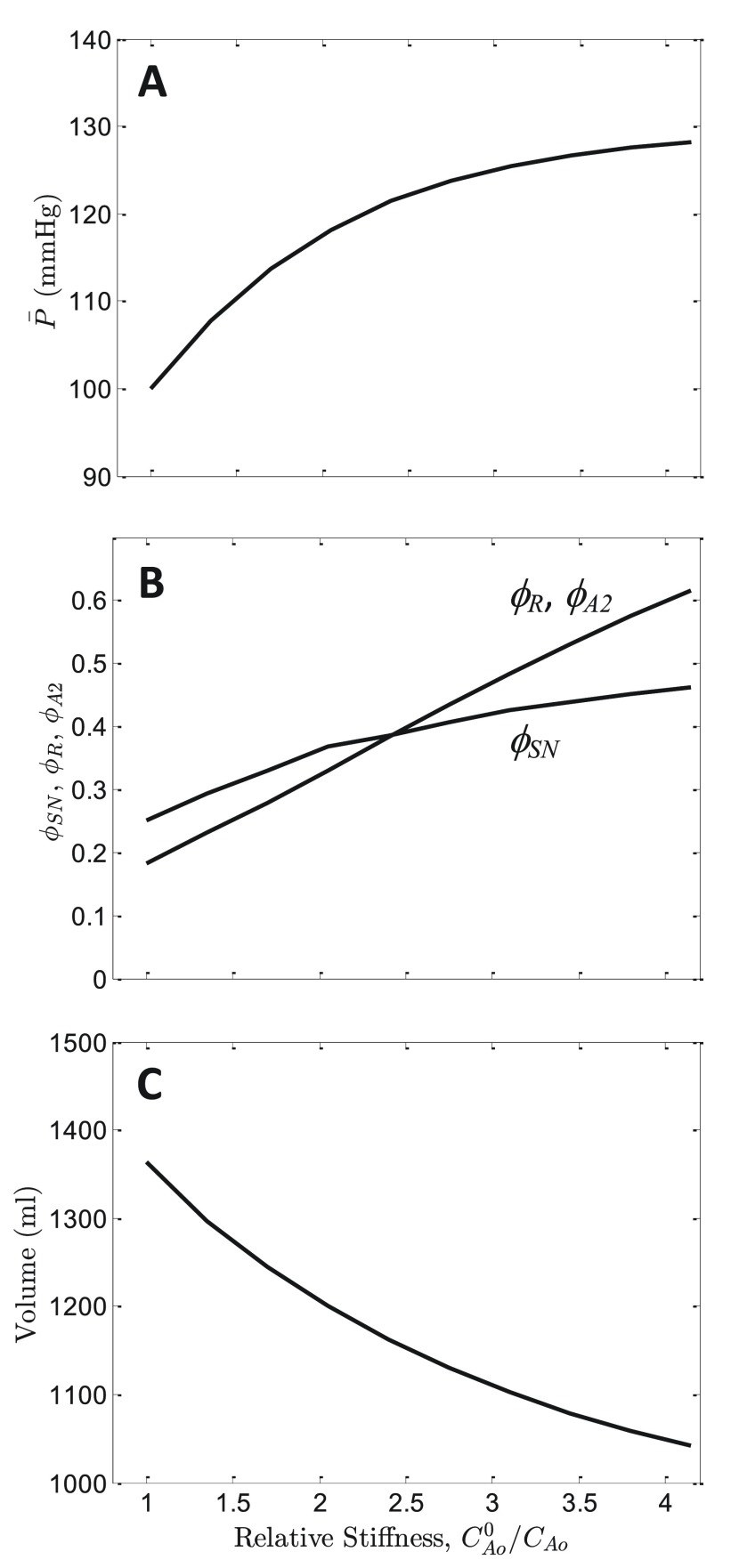
Effects of arterial stiffening on mean pressure. **A**. The model predicted for the steady-state mean arterial pressure is plotted as a function of relative aortic stiffness, *C _Ao_* ^0^/ *C _Ao_*, where *C _Ao_* ^0^ is the baseline normal value of aortic compliance, and *C _Ao_* is the value used to obtain the simulated pressure. As stiffness is increased (as compliance is decreased), predicted mean pressure increases. Calculations assume normal salt/volume loading, *Q _in_* = 0.5835 ml min ^-1^, resulting in a mean pressure of 100 mmHg at *C _Ao_* ^0^/ *C _Ao_* = 1. **B**. Model-predicted steady-state sympathetic tone *ϕ _SN_*, plasma renin activity *ϕ _R_*, and angiotensin-II activity *ϕ _A2_* are plotted as functions of *C _Ao_* ^0^/ *C _Ao_*. **C**. Predicted blood volume is plotted as a function of *C _Ao_* ^0^/ *C _Ao_*.

Using the current model, which does account for blood volume regulation by the kidneys, the hypothesis of Pettersen *et al.* may be further analyzed by determining how simulations of the current model respond to changes in the aortic compliance. Figure 10A (solid line) plots predicted steady-state mean arterial pressure as a function of relative aortic stiffness, *C _Ao_* ^0^/ *C _Ao_*, where *C _Ao_* ^0^ is the baseline normal value of aortic compliance, and *C _Ao_* is the value used to obtain the pressures reported in the Figure. The maximum simulated relative stiffness, *C _Ao_* ^0^/ *C _Ao_* = 4, is approximately the average relative stiffness for the 75-year-old population simulated in Pettersen *et al.* ^29^. This 4-fold increase in aortic stiffness increases the predicted mean pressure from 100 to 128 mmHg, with systolic/diastolic ratio increasing from 115/90 to 139/117. Furthermore, over the simulated range of stiffness model-predicted *ϕ _SN_* increases from 0.25 to 0.45 and *ϕ _R_* increases from 0.186 to 0.59 ( Figure 10B) with a heart rate increase of approximately 25% as mean pressure increases from 100 to 128 mmHg.

As expected, this predicted pressure increase is less than that predicted for a model that does not account for blood volume regulation. (The model of Pettersen *et al.* predicts a mean arterial pressure 150 mmHg for 75-year-old group). The difference between the predictions of the current model and that of Pettersen *et al.* is explained primarily by blood volume regulation: with a 4-fold increase in aortic stiffness the current model predicts the active blood volume to decrease from 1364 to 1050 ml ( Figure 10C). This result supports the view that normal functioning kidneys are able to partially compensate for age-related increases in arterial stiffness ^29^.

Therefore this model demonstrates that it is possible to capture the age-related phenotype of primary hypertension as emerging solely from changes to the mechanical properties of the aorta and carotid arteries. This may seem surprising since the simulation results plotted in Figure 10 are obtained with mechanical properties of all components of the vasculature other than large vessels associated with the baroreflex remaining normal. Indeed, it is expected that vascular stiffening would directly impact other physiological processes. For example, Beard and Mescam demonstrated that stiffening of renal arteries can explain the shift in the acute pressure-diuresis curve associated with changes in mean pressure in Dahl S rats ^31^. For the model results illustrated in Figure 10 (representing normal kidney function), the predicted increase in mean pressure associated with increased vascular stiffness is partly due to an increase in the pressure that is required to elicit a given baroreceptor firing rate. As a result, the relationship between baroreceptor firing and mean arterial pressure is shifted to higher pressure in hypertension, as has been observed clinically and in animal models ^7^. This phenomenon has been called “adaptation” or “resetting”. Yet, even though model predictions are in agreement with the observed adaptation of the baroreflex, model predictions are not in agreement with the interpretation that, since baroreceptors adapt, “they cannot participate in the long-term control of arterial pressure” ^7^. By contrast, the observed adaptation is a crucial component of the mechanogenic mechanism simulated here. Because stiffening causes a long-term adaptation of the relationship between pressure and baroreceptor firing rate, vascular stiffening can cause a chronic increase in stable mean pressure. In other words, since baroreceptors adapt, they can contribute to the etiology of hypertension.

These model predictions are in agreement with experimental observations of Thrasher, who has shown that mechanically suppressing baroreceptor sensitivity leads to sustained elevation in arterial blood pressure in a canine model ^32, 33^. Our model predictions are consistent with Thrasher’s conclusion that mechanical unloading of baroreceptors leads to sustained increases in arterial pressure ^34^.

## Parameter sensitivity

Sensitivity values of parameter estimates were estimated by computing the relative change in the mean-square error difference between data and model simulation associated with a change in the parameter value. For example, as explained above, parameters *τ _s_*, *δ*_0_, *f*_0_, *a*, and *b* were estimated by matching model predictions to data in Figure 2A and Figure 3. If *E* is the minimum normalized mean-square error difference between model prediction and data, then the sensitivity associated with parameter *τ _s_* is estimated as | *E*( *τ _s_* + 0.1 *τ _s_*) - *E*( *τ _s_*) | /0.1. Thus a sensitivity value of 1 for a given parameter would mean that a 10% change in the parameter is associated with a 10% in the error. Sensitivities for all parameters identified based on the data in Figure 1– Figure 3, Figure 6– Figure 8 are listed in Table 1. Computed sensitivity values range from 0.05 (for *α*_2_) to greater than 100 (for *C _Ao_*).

The low sensitivity of results to *α*_2_ can be understood by examining the expression of *R*_A_( *t*) in Equation (13). Since the estimated value of *α*_2_ is greater than 10, the expression is effectively approximated by *R _A_*( *t*) = *R _A_*_0_ *α*_2_ *ϕ _SN_*(1 + *α*_4_ *ϕ _A2_*), where the constants *R*_A0_ and *α*_2_ cannot be separately identified. Since the baseline resistance and compliance parameters (including *R*_A0_) are adjusted to (for given values of *α*_1_, *α*_2_, *α*_3_, and *α*_4_) are adjusted so that the model generates the reported baseline pulse pressure, cardiac output, and ejection fraction, the value of any of *α*_1_, *α*_2_, *α*_3_, and/or *α*_4_ becomes arbitrary for values significantly greater than 10.

## Summary and conclusions

A computational model (Source code is permanently available on: http://zenodo.org/record/7126 ( 10.5281/zenodo.7126)) was developed and identified to serve as a phenomenological representation of the major physiological processes controlling arterial blood pressure. The design of the model was based on balancing the compromise between complexity/physiological fidelity and identifiability, yielding a model of 16 state variables and 46 parameters. Of the 46 parameters, 23 were set to previously established values or arbitrary values as explained above, and 23 were adjusted to match experimental data illustrated in Figure 1– Figure 3, Figure 6– Figure 8. The model captures physiological phenomena occurring on times scales ranging from milliseconds (e.g. response of baroreceptor firing to arterial pressure) to days (e.g. response of mean pressure to chronic stimulation of baroreflex). It also effectively captures the response of the cardiovascular system to drastic perturbations, including infusion of 45% of blood volume, withdrawal of 35% of blood volume, electrical stimulation of the baroreflex, and the partial compensatory response of the kidneys to blood pressure increase accompanying arterial stiffening.

The major conclusions from this work are:

1. The observed adapting/resetting of the baroreflex to long-term changes in pressure does not indicate that changes in baroreflex function cannot play a role in long-term changes in arterial pressure. Model simulation reveals how the baroreflex arc and the renin-angiotensin system may both contribute in a coordinated manner to affect long terms changes in arterial pressure.

2. It is demonstrated how renal function is not the central or primary determinant of long-term arterial pressure in a computer model that captures the observed behavior of the renin-angiotensin system, and chronic adaptation of the acute pressure-diuresis/natriuresis phenomena. This result contradicts the classical ‘renocentric’ view of the long-term control of blood pressure even though the model is built on the core assumption of the renocentric view that the pressure-diuresis relationship acts as a physiological input-output relationship in which pressure determines renal output. As a result, it is shown that hypertension does not necessarily indicate any degree of renal dysfunction.

3. The physiological response to chronic stimulation of the baroreflex can be explained by a mechanism in which arterial blood pressure is regulated primarily through the baroreflex arc and the interoperation of the baroreflex arc and the renin-angiotensin system.

4. Model simulations are consistent with the mechanogenic hypothesis ^29^ that arterial stiffening represents a contributing factor causing changes in pressure, sympathetic tone, and renal function associated with ageing. Through pressure-diuresis, normal kidney function is predicted to be able to partially ameliorate the effects of arterial stiffening on blood pressure.

Regarding the forth conclusion, model simulations predict that stiffening of the large arterial vessels that are associated with baroreceptors can contribute substantially to long-term changes in pressure. This result contradicts the ‘renocentric’ theory of blood pressure control even though the core assumption of the Guyton ‘renocentric’ theory of blood pressure control (that the pressure-diuresis relationship acts as a physiological input-output relationship in which pressure determines renal output) is a core assumption of this model. Although it has been argued that because its effective gain resets to a given mean arterial pressure the baroreflex cannot play an important role in long-term pressure regulation, this analysis shows how a dynamic system representing the baroreflex and capturing the observed resetting phenomenon can play a key role in the long-term control of pressure and the etiology of hypertension. Model simulations reveal that vascular stiffening increases sympathetic tone and shifts the effective baroreflex response to an increased pressure baseline. Because these changes cause an increase in angiotensin II activity and associated shift in the acute pressure-diuresis relationship, the renocentric ^7^ and mechanogenic ^29^ hypotheses for the etiology of primary hypertension could potentially be interpreted as compatible. However, as changes to renal function arise as downstream consequences of the mechanical remodeling, the mechanogenic explanation subsumes more of the biology involved in the etiology of hypertension compared with a renocentric explanation. This is a major conceptual advance that also provides a new interpretational framework for available experimental and clinical data.

The model developed here represents a simplification and several components are represented largely in phenomenological terms. A more detailed model may be constructed through integration of more biophysically based and detailed component models ^30, 31, 35^ into the framework developed here. Regardless, the simplifications invoked in the model do not impact the major conclusions. Perhaps as important as the simplified modeling framework is the fact that a model identified from data obtained from experiments on dogs and rabbits, is used to draw insight into human physiology and pathophysiology. The implicit assumption here is that arterial pressure regulation processes are at least qualitatively similar in dog, rabbit and man.
