# Supplementary material for: A computational analysis of the long-term regulation of arterial pressure
Source: F1000Res. 2013 Dec 6;2:208. Originally published 2013 Oct 8. [Version 2] doi: 10.12688/f1000research.2-208.v2 (PMC3886803; doi:10.12688/f1000research.2-208.v2)
Supplement: Peer review history

# Peer review: Referee response for version 2

**Published:** 2013-12-12

**Authors:** Patrick Hannaert

**Affiliations:** 1 INSERM U1082, Poitiers, France

The authors acutely revised the manuscript, provided convincing explanations/comments, answered key issues, and nicely complemented their work.

I have read this submission. I believe that I have an appropriate level of expertise to confirm that it is of an acceptable scientific standard.

# Peer review: Referee response for version 2

**Published:** 2013-12-10

**Authors:** John Osborn, Viktoria Averina

**Affiliations:** 1 Department of Integrative Biology and Physiology, University of Minnesota, Minneapolis, MN, USA; 2 University of Minnesota, Minneapolis, MN, USA

The authors have done an excellent job responding to our comments.

We have read this submission. We believe that we have an appropriate level of expertise to confirm that it is of an acceptable scientific standard.

# Peer review: Referee response for version 2

**Published:** 2013-12-09

**Authors:** Johnny Ottesen

**Affiliations:** 1 Department of Science, Systems, and Models, Roskilde University, Roskilde County, Denmark

I find the paper worth reading as it present new ideas. In the revision process it has even been improved.

I have read this submission. I believe that I have an appropriate level of expertise to confirm that it is of an acceptable scientific standard.

# Peer review: Referee response for version 1

**Published:** 2013-11-26

**Authors:** Patrick Hannaert, François Guillaud

**Affiliations:** 1 INSERM U1082, Poitiers, France

**SUMMARY**

**This work by Beard *et al*., is an interesting, consequential and mind-tickling work, definitely worth considering and being published, provided that several issues are addressed.**

**Strong points:**

- **Sound “horizontal” (multi-organ) physiological modelling & simulation (following a systems/engineering analysis and conception).**
- **Modular, integrative, multi-dynamic approach.**
- **Targeted, specific hypothesis - proposes one possible/sufficient etiogenic mechanism in the context of age–related hypertension.**

**Weak points:**

- **Phenomenological equations.**
- **Some conclusions drawn are too assertive (especially with respect to such a complex, multi-factorial, and evolving, pathology: hypertension); the likeliness of the conclusion appears strongly dependent on the limited physiology (and pathology) that is implemented.**

**1. GENERAL**

In bio-medical research, it has become understood that mathematical modelling and simulation (MS) can provide a complementary approach to experiments and are a necessary response to extreme biological complexity. Indeed, biochemical and genetic circuits modelling are becoming mature and productive. On the other hand, there remains a very serious “modelling gap” to be filled between the (“low-level”) biological/physiological processes and (“higher-level”) pathophysiology.

Simply put, the “CV-circulatory-pressive” construct by Guyton and colleagues (started in the late 60’s, with 1972 and 1992 milestones; Guyton *et al*., 1972; see Montani & Van Vliet, 2009, Moss *et al.,* 2012) remains a landmark, to the point that most, if not all modelling endeavours refer to it in one way or another. This is because it was an exhaustive attempt to describe the CV circulatory system in its physiological entirety, including high-level interactions such as an integrated CV and renal system.

Many versions, declinations (and improvements) do exist, including the late HumMod ( Hester *et al, *2011 should perhaps be cited, instead of Abran *et al.*, 2007, since it is the current evolution of QCP/QHP). However, as mentioned by the Authors, for various reasons it is difficult or impossible to rationally understand and exploit those models (e.g. “too large” in the case of HumMod, or “not identified” as in the case of Guyton’s models of 1972 or 1992).

Beard *et al.* propose here a “top-down”, modular, “engineering” high-quality MS work, as a rationally exploitable (numerically identifiable) alternative to Guyton’s model, in order to explore hypertension etiogenic hypothesis related to arterial stiffness, baroreflex and the renin-angiotensin system (RAS). The main incentive relates to the controversed Guytonian view that long-term regulation of arterial pressure is driven by the kidney. In that context the Authors try to demonstrate that arterial stiffening (as in ageing) and/or the central nervous system (here simplified as the “baroreflex”) could provide a sufficient explanation(s) of hypertension, through interactions with the kidney pressure-natriuresis function and the RAS.

The significance and relevance of this work for CV regulations and hypertension is obvious, although

(i)  it is limited by the model itself (i.e. effectively implemented features, simplifications, phenomenological approach for equations, etc.) and

(ii) some of the conclusions/assertions should be tempered (among other reasons: given the multifactorial/multi-dynamic nature of HT, that it has been resistant to investigation for more than a century now, and given the simplicity of the proposed construct).

 

**2. MAJOR COMMENTS**

In the targeted domain of the pathophysiology of blood pressure regulation and primary hypertension, this work proposes a rigorously constructed and parameterized mathematical model of the *“long-term control of arterial pressure”* (but see below). The authors should emphasize however that the model numerical instantiation centers on dog (or rabbit). Also of important note (this should be addressed in the revised version), no mention is made of the parameterization procedure and tools or sensitivity analysis.

Moreover, the parameterization is done under the (quite frequent) implicit hypothesis that arterial pressure regulation and other CV-related processes only differ quantitatively (e.g. in parameter values) and are otherwise qualitatively similar e.g. in dog, rabbit and man. This very point is rarely, if ever, addressed. We do feel that in the present turmoil of “physiological” models targeting *human* pathology, this should be, at minimum, mentioned and preferably justified. Given that the Authors target *human* hypertension, and especially given the phenomenological approach chosen, why did they not directly parameterize their model as a “human” CVR model?

As a matter of fact, the anatomy and mechanical/physiological/biochemical properties and functions of the human CV system, arteries, heart, kidney and RAS are, to the best of our knowledge, now understood and numerically characterized enough to allow for such a human quantitative approach to be adopted (especially with this articles chosen phenomenological “input-output” approach). This would have given some more resonance and relevance to the work. In particular, even if all CV-pressive, renal and hormonal processes proved to be the same between all these species, the possibility would remain that their relative contributions (and dynamics) to arterial pressure (dys-) regulations are different.

As their objectives, the Authors aimed to:

1. develop a *"computer model capturing chronic adaptation of acute renal function in blood pressure control"* as a more rigorous alternative to  Guyton’s  circulatory model ( Guyton *et al., *1972) and to Guyton’s-derived approaches, particularly SAPHIR ( Thomas *et al., *2008, - Hernandez *et al.,* 2011, could be cited too here, as well as Moss *et al.,* 2012)
2. thus *"capturing the relevant mechanisms".*

These are very strong and ambitious objectives, and possibly *ab initio *flawed by the phenomenological (but otherwise legitimate) approach chosen.

Further, that this is *“a model capturing chronic adaptation of acute renal function in blood pressure”* cannot be claimed with such a simple kidney function, for example: the pressure-natriuresis ignoring actions of the ANS (autonomic nervous system) upon renal hemodynamics and tubular function and the angiotensin action on glomerular and tubular function (for instance Glomerular Tubulo Balance and Na/water reabsorption). Kidney nerve *afferents* are also ignored.

What is really addressed in this work are the interactions (possibly etiogenic) between the physiological (input/output) modules that are implemented.

Finally, after more than a century of physiological and clinical research, the etiology of primary hypertension remains unresolved; although it is established that (numerous), genetic, environmental, age-related and adaptive factors do intervene. Moreover, when in the realm of age-related hypertension the Authors do select putative etiogenic processes (arterial stiffening, reduced BR receptors sensitivity) and explore potential interactions with RAS and kidney function (phenomenological pressure-natriuresis) in a simplified CV construct.

 

Driven by the objective (age related HT/arterial stiffening), the proposed model features six central physiological functionalities, as modules (model components) implementing:

MC1. “Aorta-large artery mechanics”

MC2. “Kinetics of baroreflex afferent firing”

MC3. “Mechanics of the heart and circulation”

MC4. “Autonomic system”

MC5. “Neuro humoral control of pressure diuresis/natriuresis”

MC6. “Renin-angiotensin system”

 

For each one of these model components (physiological organ/process), the Authors should give, where possible, at least one published “first-principle” (physiologically-based) model as a reference, e.g. amongst others (i) Moss and Thomas, 2013 for the kidney/pressure-natriuresis under hormonal influence, (ii) Guillaud and Hannaert, 2010 and/or Claassen *et al.*, 2013 for the RAS.

 

The model is first validated (short-term) vs haemorrhage and then volume expansion. Of note, no real validation criterion is given (except for visual adequation through the figures).

Secondly, the model is validated (long-term = days to weeks) using published steady-state pressure-natriuresis kidney function data. Although the Authors target long-term processes, 2 weeks (maximal simulated time here) is far too insufficient to refer to the CV, renal and central physiological mechanisms truly involved (months-to-years in dogs, years and decades in man).

 

**OTHER COMMENTS**

- Is one of the input arrows missing in the model figure (fig 4)?
- A model diagram (rather than the electrical analog presented), as well as for the main hypothesis (vs alternative(s)) would be welcome.
- To what extent does this work differ from the previous work by the same group (cited, see Pettersen *et al.,* 2013)?
- The Matlab(C) model code and scripts proposed ( http://zenodo.org/record/7126#.UpOLNOJ21pg) have been tested and verified by Dr F. Guillaud (ModTeam/Inserm U1082). The scripts could be run, the model could be simulated and results that were visually identical to those in the paper were obtained.

We have read this submission. We believe that we have an appropriate level of expertise to confirm that it is of an acceptable scientific standard, however we have significant reservations, as outlined above.

# Response

**Published:** 2013-11-26

**Authors:** Daniel Beard

**Affiliations:** University of Michigan, USA

The reviewers have provided an extensive and insightful commentary on our paper. We agree with most of the points raised. We have extracted specific criticisms and responded to them below.

 

**Weak points:** **Phenomenological equations;**

 

This point is addressed in some detail in the responses to the first two reviewers, as well in response to specific points below.

 

**Many versions, declinations (and improvements) do exist, including the late HumMod (** **Hester *et al, *2011** **should perhaps be cited, instead of Abran *et al.*, 2007, since it is the current evolution of QCP/QHP).**

 

The more recent citation has been inserted into the revised text.

 

**...some of the conclusions/assertions should be tempered...**

 

The validity of our conclusions is addressed extensively in our response to the first set of reviewers. In brief, we agree that some statements needed to be clarified and rephrased. See responses above for details.

 

**The authors should emphasize however that the model numerical instantiation centers on dog (or rabbit); the parameterization is done under the (quite frequent) implicit hypothesis that arterial pressure regulation and other CV-related processes only differ quantitatively (e.g. in parameter values) and are otherwise qualitatively similar e.g. in dog, rabbit and man.**

 

This is an important point that cuts across all areas of computational physiology. Large-scale models are built based on data from multiple sources, multiple species, different conditions (in vitro, in vivo, etc.) In the end, all of our models are chimeras. We have taken the liberty of adapting the reviewers’ language at the end of the discussion section of the revised paper. (We do point out that our implicit assumption is the central assumption of all biomedical research using animal models. It might not be a perfect assumption, but it does give us a starting point.)

 

**Also of important note... no mention is made of the parameterization procedure and tools or sensitivity analysis.**

 

Please see our detailed response to this point in the response to the first review.

 

**For each one of these model components (physiological organ/process), the Authors should give, where possible, at least one published “first-principle” (physiologically-based) model as a reference**

 

This is an excellent idea in principle. However, it is not clear if it is possible. For example, we are unclear as to how the Moss and Thomas paper could be characterized as representing the hormonal influence on pressure-natriuresis from first principles. Specifically, the Moss and Thomas paper represents the action of angiotensin-II “ *via the parameter FrANG, which adjusts the response of the pressure-natriuresis mechanism.*” The parameter FrANG appears in the model equations as a factor multiplying a phenomenological expression for the fractional reabsorption, and the effect of the hormone is simulated by varying FrANG between “ *a maximal value of 1 (minimal angiotensin II) and a minimal value of 1/6 (maximal angiotensin II)*”. Thus, the model is every bit as “phenomenological” as the one presented here. (This point is not to be taken as a criticism of the excellent study of Moss and Thomas.)

 

The papers by Guillaud and Hannaert, and Claassen et al. do account for the RAS system with more physiological fidelity/detail than our model. We do disagree with the assertion that any of the models discussed here are built from “first” principles. All of these papers invoke the same sort of phenomenological expressions for certain components.

 

**Although the Authors target long-term processes, 2 weeks (maximal simulated time here) is far too insufficient to refer to the CV, renal and central physiological mechanisms truly involved...**

 

The reviewers may be correct that our model is far too insufficient to provide insight into a pathophysiological mechanism that emerges over decades. Yet, we refer the reviewers (and the readers) to the stated conclusions in the paper. 

 

**Is one of the input arrows missing in the model figure (fig 4)?**

 

The net volume output rate is Q_urine – Q_in. The figure has been revised to clarify this.

 

**A model diagram (rather than the electrical analog presented), as well as for the main hypothesis (vs alternative(s)) would be welcome.**

 

This has been added. (See Figure 4B.) The main hypothesis here is that pressure-diuresis acts as a physiological input-out relationship, and that renal function is controlled through the RAS and the autonomic system. Thus, fundamentally, the model is constructed to capture the Guyton concept of the long-term control of arterial pressure. (Therefore, it is not clear what alternatives we might indicate in a model diagram.)

 

**To what extent does this work differ from the previous work by the same group..?**

Please see the Section “Results 5: Exploration of the etiology of primary hypertension” in the revised text.

# Peer review: Referee response for version 1

**Published:** 2013-11-14

**Authors:** Johnny Ottesen

**Affiliations:** 1 Department of Science, Systems, and Models, Roskilde University, Roskilde County, Denmark

Complex physiologically systems are often subdivided into parts and studied separately by experimental methods. But in experimental physiology, subsystems are not easily put back together and conclusions made concerning subsystems have limited validity for the original undivided system as their function and dynamics may have changed when isolated from one another. Parts that cannot be isolated experimentally may be studied (separately) using modeling. Mathematical modeling is able to unfold the influence that each of the processes has on the overall dynamic and thus modeling is highly adept at separating systems into components simple enough for their structures and functions to be studied in isolation. Mathematical modeling is the *only controlled *way to put the pieces back together by using equations that represent the system's components and processes, as well as its structures and interactions.

 

In *“A computational analysis of the long-term regulation of arterial pressure”* the authors follow this strategy nicely in a successful attempt to investigate what they denote the *‘renocentric’* theory by Guyton. Here the authors combine cardiovascular and baroreflex models with models of the renin-angiotensin system whereby some important conclusions are reached. Among these conclusions the authors emphasize that the baroreflex may play a key role in the long-term regulation of the arterial pressure and especially in hypertension in contrast to the Guytonian view.

 

The authors follow the physiologically structure well in their models as they carefully validate the different sub-models/elements by comparing them with existing various animal experiments. These sub-models are then integrated into a well-behaved and solid overall model.

 

However, there are some methodic drawbacks I would like to emphasize: Most model equations are in some sense ad-hoc choices (or phenomenological, as the authors mention at the very end). These equations are not based on first principles and hence they do not represent the underlying mechanisms. Instead the approach is to assume a first order transient toward some static sigmoidal relationship. In addition, the authors make a lot of self-references, which shadows the fact that many of the used sub-models are adjustment taken from elsewhere and ideas from other sources. Strengthening the paper with such information would definitely support the approach taken.

 

The paper is very well structured and written but a few misprints have sneaked in:

 

Formula (3): Should the fraction be / *C*_Ao_ instead of *C*_Ao_/?

Formula (4): An index o is missing on V _sA_

On page 4, coulomb 2, line 2:  should be

On page 4, coulomb 2, line 5: *‘… the population transition from an active state at a rate …*’ Please state reference (or state that this is a hypothetical assumption)!

I have read this submission. I believe that I have an appropriate level of expertise to confirm that it is of an acceptable scientific standard.

# Response

**Published:** 2013-11-26

**Authors:** Daniel Beard

**Affiliations:** University of Michigan, USA

Thank you for the attention to our paper and the constructive criticisms.

 

**Most model equations are in some sense ad-hoc choices (or phenomenological, as the authors mention at the very end). These equations are not based on first principles and hence they do not represent the underlying mechanisms... In addition, the authors make a lot of self-references...**

 

We agree with the reviewer’s assessment. To reiterate a statement from the paper summary, the model represents a balance complexity/physiological fidelity and identifiability. The sensitivity analysis added to the revised paper indirectly addresses the issue of the phenomenological nature of model components.  Finally, we have revised the text to clearly state the nature of the model earlier than the last paragraph of the paper. See third paragraph on page two, section on “Model Components 5”, and first paragraph of the summary.

 

Also, we point out that while we have cited some previous modelling works, the majority of components used here have been constructed *de novo*, with the overall goal in mind. The varying elastance heart model is adapted from the cited reference. And of course there is nothing new about lumped-parameter models of the circulation. But most of the other (phenomenological) formulae are constructed to match the observed dynamics of responses of these systems to the experiments used to identify the model.

**Formula (3): Should the fraction be / *C*_Ao_ instead of *C*_Ao_/?**

No, it is correct in the paper. One way to see this is to think about the limit where *C*_Ao_ goes to infinity. In this case the vessel becomes infinitely compliant if we wait long enough and thus does not support any finite pressure.

**Formula (4): An index o is missing on V _sA_**

 

Fixed.

**On page 4, column 2, line 2:  should be d _0_**

 

Fixed

**On page 4, column 2, line 5: *‘… the population transition from an active state at a rate …*’ Please state reference (or state that this is a hypothetical assumption)!**

 

The assumption of Equation (7) is stated: “ *It is assumed that the baroreceptors within the population transition from an active to inactive state at a rate proportional to the firing rate*”. We do not know what factors are responsible for the slow “resetting” of the baroreflex response. But, it is compelling that this simple equation is able to capture the nonlinear phenomena of Figures 2 and 3.

# Peer review: Referee response for version 1

**Published:** 2013-11-04

**Authors:** John Osborn, Viktoria Averina

**Affiliations:** 1 Department of Integrative Biology and Physiology, University of Minnesota, Minneapolis, MN, USA; 2 University of Minnesota, Minneapolis, MN, USA

This paper by Beard *et al* makes three major claims: 1) to provide a mathematical description for Guyton’s existing model; 2) to show how stimulation of the baroreflex arc can explain long-term changes in blood pressure via the renal function curve employed in Guyton’s model; and 3) to show how arterial stiffening can lead to hypertension without renal injury. I commend the authors for making a much needed mathematical contribution to the field of computational physiology, where one of the most widely recognized and used models of long-term control of arterial pressure has only very rudimentary mathematical description and analysis published. Moreover, the authors are bridging the gap between the renocentric view of hypertension etiology and the emerging experimental data that points to an independent long-term role of neural regulation. For these reasons the paper is a valuable addition to the existing body of knowledge.

There are several issues however, that need to be addressed in a revised version of the paper. The overarching recommendation is to correct the paper’s focus and claims to make them more consistent with the model design, as well as to make the model’s description easier to understand. In particular, I believe the model should not be presented as a generic model with the ** *“representation of the major physiological processes controlling arterial blood pressure”* but instead be positioned as a conceptual framework which aims to illustrate how the baroreflex arc may be involved in long-term blood pressure control. Phenomenological mathematical representation, a small amount of detail, limited analysis, and quality of shown simulations are appropriate for a conceptual model but are insufficient for a model from which one could be extracting conclusions on the particular physiological mechanisms to be implicated in hypertension development.

**Suggested revisions:**

There are several versions of Guyton’s model ranging from very simplistic to very complex ^1^. Some models are extremely detailed but have a closed architecture (e.g. hummod.org). The model created by the authors is not sophisticated enough to correspond to most of these versions. Please state clearly which version of Guyton’s model is being considered here. In my opinion, the model proposed here seems of approximately the same level of complexity to the one presented by Guyton and Coleman *'Long-term regulation of the circulation: interrelationships with body fluid volumes' *in the text *Physical bases of circulatory transport: Regulation and exchange*. (1967) ^2^

1. Focus the goals according to what was accomplished in the paper:
  - *“capturing the relevant mechanisms in an identifiable mathematical model”* is too over-reaching since the three key equations (18, 19, and 21) do not capture mechanisms but are of phenomenological nature.
  - *“probing hypotheses regarding the long-term control of arterial pressure”* – the model is both too limited and phenomenological to allow for any testing of various hypotheses. It is, however, fit for providing a possible explanation for two hypotheses: roles of the baroreflex involvement and arterial stiffening. Otherwise, for testing, one would require a more mechanistic description, inclusion of other key organs and regulatory mechanisms, and a more rigorous mathematical analyses. 
2. The authors provided an explanation of how the baroreflex may be involved in long-term control of blood pressure. This is a novel contribution to the field and should be the focus of the discussion:
  - Add the discussion and references of experimental knowledge of baroreflex stimulation, inhibition, and especially long-term resetting.
  - Describe how Guyton’s models incorporate neural control and explain the above finding, especially the effects of chronic stimulation and how it is different from the proposed model (e.g. ^3^)
  - Discuss existing knowledge or a possible physiological explanation for the finding that renin activity will be diminished with baroreflex stimulation.
  - Discuss what other important physiological mechanisms are not reflected in the model and how they may play a role. In particular, explain why macula densa sodium delivery is thought not to be important enough to include in the model, despite the fact that it is known to be one of the key regulators of renin activity.
  - Do not confuse the model’s predictions with its assumptions when discussing simulation results. The model assumes and is explicitly designed to tie the baroreflex, renin, and SNA with long-term pressure control. An explicitly designed assumption is not the same as prediction. Thus, statements like Conclusion 1 in “Summary and Conclusions” are inappropriate. The fact that the model’s assumptions allow for physiologically plausible simulations merely supports the notion that baroreflex may be important in the way the authors have described it.   
3. Improve the clarity of the mathematical description: 
  - Provide motivation for the chosen elements of the model and outline the model in its entirety first. For example, a flowchart or a diagram of the model may help readers to grasp the overall idea.
  - Add at least a basic analysis of parameter sensitivity, with clear description in the Methods section.
  - In Table 1 please provide physiologically-based description of parameters rather than referring to the equations where they are used (e.g. Emax is the maximal left-ventricular elastance, rather than *“Varying elastance heart model, Equation (10)”*). Otherwise, the definitions are circular: parameters are used in the equations without an explanation of their meaning in either the text or the table.

We have read this submission. We believe that we have an appropriate level of expertise to confirm that it is of an acceptable scientific standard, however we have significant reservations, as outlined above.

## References

[1] GuytonACColemanTGGrangerHJ: Circulation: overall regulation.*Annual Review of Physiology.*.1972;34: 20813-4410.1146/annurev.ph.34.030172.0003054334846

[2] GuytonACColemanTGGranger: Lowering of blood pressure during chronic suppression of central sympathetic outflow: insight from computer simulations.*Physical bases of circulatory transport: Regulation and exchange*.1976;

[3] IlliescuRLohmeierTE: Lowering of blood pressure during chronic suppression of central sympathetic outflow: insight from computer simulations.*Clinical and Experimental Pharmacology and Physiology*.2010;37(2) : 20810.1111/j.1440-1681.2009.05291.x.19769610PMC3872032

# Response

**Published:** 2013-11-26

**Authors:** Daniel Beard

**Affiliations:** University of Michigan, USA

We are grateful to the reviewers for paying such serious attention to our paper and offering many constructive criticisms. While we have revised the paper to address the concerns and clarify the presentation, we disagree with the general interpretation of our model as “ *a conceptual framework which aims to illustrate how the baroreflex arc may be involved in long-term blood pressure control.*” As stated in the summary, the model “ *was developed and identified to serve as a representation of the major physiological processes controlling arterial blood pressure*.” For example, there is no equation or parameter value artificially injected into the model to require pressure-diuresis to not be able to return the pressure to a baseline value during baroreflex stimulation. In fact, we could parameterize the model so that the chronic pressure-diuresis phenomenon acts as an “infinite-gain” control system, always returning pressure to its set point. But in doing so, the model would not be able to match the data used to identify it, or to predict the data used to validate it.

 

Regarding the level of detail captured by the model, the reviewers are correct that at some level, all components of the model are phenomenological in nature. (This is true of all mathematical models.) Yet regarding the question around the level of detail and complexity of the model, we stand by the assertion that this model represents the most detailed model of long-term control of arterial pressure that is identified in the literature. (Here we are invoking the word “identified” to indicate that the model equations and associated parameter values are reported along with comparisons of model simulations of data used to identify the parameters.)

 

In fact, one of the unstated goals of this paper is to inject some clarity and rigor into this field with precise and concrete language. Therefore we do not know how to respond to the statement that our model “ *is not sophisticated enough to correspond to most of*” the versions of the Guyton model because it is not clear, precisely, what is meant by “sophisticated” or “correspond”. In discussing the Guyton models, we clearly state that: “ *The 1972 realization of the model invokes approximately 160 variables, and several hundred adjustable parameters*. *More recent versions of the model involve many thousands of variables and tens of thousands of parameters*.” Although it is true that our model has less variables and parameters than any version of the Guyton model that we are aware of, is the sophistication of a model measured purely by the number of variables or adjustable parameters? Since no version of the Guyton model captures pulsatile mechanics, no version of the model can capture the baroreceptor resetting phenomena illustrated in Figures 2 and 3 of our paper. Nor does the HumMod model capture the system response to baroreflex stimulation illustrated in Figure 9. Therefore one could argue that our 16-variable model is more sophisticated than the 5,000-variable Guyton model.

 

Regarding the specific suggestions:

 

**“capturing the relevant mechanisms in an identifiable mathematical model” is too over-reaching since the three key equations (18, 19, and 21) do not capture mechanisms but are of phenomenological nature.**

 

More specifically, we stated that one of the “goal” of the study is “ *capturing the relevant mechanisms in an identifiable mathematical model*.” We believe we have done that. However, in the revised paper we have changed the goal to “ *representing the relevant mechanisms in an identifiable mathematical model*.”

 

**“probing hypotheses regarding the long-term control of arterial pressure” – the model is both too limited and phenomenological to allow for any testing of various hypotheses.**

 

Again, we counter that we have met the stated goal. We are not claiming to have proved any hypothesis. Yet we have introduced some important disproofs, as outlined in some of our major conclusions. Specifically, it is stated in the literature as ‘scientific facts’ that; the renal function curve represents the dominant long-term controller of blood pressure and, that since the baroreflex “resets” it cannot play a role in chronic changes in mean pressure. Our model analysis has cast significant doubt upon the validity of both of these hypotheses. Admittedly, these findings represent somewhat of a double negative i.e., a model capturing baroreflex resetting is used to demonstrate how a baroreflex that resets could play a role in long-term changes in pressure. Therefore, we have shown that the fact that the baroflex resets, does not mean that the baroreflex cannot play a role in long-term pressure control. Therefore we are confident that it is appropriate to state that we have constructed a model with the goal of “ *probing hypotheses regarding the long-term control of arterial pressure.*”

 

**Add the discussion and references of experimental knowledge of baroreflex stimulation, inhibition, and especially long-term resetting.**

 

Data in Figures 2 and 3 represent direct experimental interrogation of baroreflex stimulation, inhibition, and long-term resetting. The resetting phenomenon is also indirectly probed in Figure 6 where, over the time course of the experiment, the baroreflex response to the acute pressure increase gradually diminishes. The phenomenon and experimental observations are discussed in association with our simulations of these experiments. These are also discussed in the section on arterial stiffening and the etiology of hypertension. Finally, we have added a couple of sentences (end of Results Section 5) comparing our model predictions with the long-term observations of Thrasher on baroreceptor unloading.

 

**Describe how Guyton’s models incorporate neural control and explain the above finding, especially the effects of chronic stimulation and how it is different from the proposed model...**

 

A discussion on this point has been added to the section “Results 4”.

 

**Discuss existing knowledge or a possible physiological explanation for the finding that renin activity will be diminished with baroreflex stimulation.**

 

It is not our finding that renin activity will diminish with baroreflex stimulation. That is the prior experimental observation. The mechanism in the model is through the observed dependence of renin release on sympathetic tone. At a deeper level, the mechanism may simply be explained as follows: decreased sympathetic tone leads to decreased renal arterial tone, leading to increased glomerular filtration and/or medullary blood flow, leading to increased flux of sodium to the macula densa, leading to decreased renin production. While we would rather leave such speculation out of the manuscript, it is published here as part of the open review process.

 

**Discuss what other important physiological mechanisms are not reflected in the model and how they may play a role... explain why macula densa sodium delivery is thought not to be important enough to include in the model, despite the fact that it is known to be one of the key regulators of renin activity.**

 

It is not that we believe that “ *macula densa sodium delivery is…not...important enough to include in the model*.” Rather the phenomenon is captured in a simplified way. To clarify this point, we have added the following text to the section on “Neurohumoral control of pressure-diuresis/natriuresis”:

 

“ *These expressions simplify the known (and unknown) mechanisms governing renin production into a simple phenomenological relationship between pressure, sympathetic tone, and the kinetics of the renin-angiotensin system*.”

 

A more biophysically detailed model would be required to represent the direct link between sodium transport and renin production. At the bare minimum, such a model would represent the biophysical processes linking pressure and solute transport in the kidney, including effects of sympathetic innervation on arterial smooth muscle stress development, mechanics of blood and filtrate flow, solute advection, diffusion, permeation, exchange, and pumping.

 

**Do not confuse the model’s predictions with its assumptions when discussing simulation results... Conclusion 1 in “Summary and Conclusions” is inappropriate. The fact that the model’s assumptions allow for physiologically plausible simulations merely supports the notion that baroreflex may be important in the way the authors have described it.**

 

We agree that this first stated conclusion does not represent a conclusion and what we are trying to say is now better captured in the revised text for Conclusion 1.

 

This same sentiment applies to the other conclusions, which are theoretical. For example, we conclude that we have demonstrated that “ *that hypertension does not necessarily indicate any degree of renal dysfunction.*” It may seem like an equivocation to insert the word “necessarily”. However, the literature is full of claims that due to the phenomenon of pressure-diuresis, hypertension necessarily does indicate some degree of renal dysfunction. Our theoretical analysis however would disprove that widespread claim.

 

**Provide motivation for the chosen elements of the model and outline the model in its entirety first. For example, a flowchart or a diagram of the model may help readers to grasp the overall idea.**

 

We have added a diagram illustrating the control processes captured by the model. (Figure 4B.)

 

**Add at least a basic analysis of parameter sensitivity, with clear description in the Methods section.**

 

This is an excellent idea. A detailed sensitivity analysis is not feasible because the model is expensive to simulate (particularly for the long time frames of Figure 9, for example), the number of parameters is large and would require calculation of 46x46 Fischer Information matrix (or cross-correlation matrix), and we do not have standard error estimates for all of the data used to identify the model. However, as suggested we have done a basic analysis, and reported a sensitivity index in Table 1. Results are discussed in a new section on parameter sensitivity.

 

(Also, the sensitivity analysis facilitated as a slightly better parameterization of the model. The results have change imperceptibly.)

 

**In Table 1 please provide physiologically-based description of parameters rather than referring to the equations where they are used... Otherwise, the definitions are circular: parameters are used in the equations without an explanation of their meaning in either the text or the table.**

 

We believe that it useful to the reader to indicate in the table where the parameter is used in the model. Therefore, the indication of which equations given parameters appear in has been retained in the table. In addition, descriptions of the meaning of all parameters have been added wherever they were missing in the text.
